# Basin-wide variation in tree hydraulic safety margins predicts the carbon balance of Amazon forests

**DOI:** 10.1038/s41586-023-05971-3

**Published:** 2023-04-26

**Authors:** Julia Valentim Tavares, Rafael S. Oliveira, Maurizio Mencuccini, Caroline Signori-Müller, Luciano Pereira, Francisco Carvalho Diniz, Martin Gilpin, Manuel J. Marca Zevallos, Carlos A. Salas Yupayccana, Martin Acosta, Flor M. Pérez Mullisaca, Fernanda de V. Barros, Paulo Bittencourt, Halina Jancoski, Marina Corrêa Scalon, Beatriz S. Marimon, Imma Oliveras Menor, Ben Hur Marimon, Max Fancourt, Alexander Chambers-Ostler, Adriane Esquivel-Muelbert, Lucy Rowland, Patrick Meir, Antonio Carlos Lola da Costa, Alex Nina, Jesus M. B. Sanchez, Jose S. Tintaya, Rudi S. C. Chino, Jean Baca, Leticia Fernandes, Edwin R. M. Cumapa, João Antônio R. Santos, Renata Teixeira, Ligia Tello, Maira T. M. Ugarteche, Gina A. Cuellar, Franklin Martinez, Alejandro Araujo-Murakami, Everton Almeida, Wesley Jonatar Alves da Cruz, Jhon del Aguila Pasquel, Luís Aragāo, Timothy R. Baker, Plinio Barbosa de Camargo, Roel Brienen, Wendeson Castro, Sabina Cerruto Ribeiro, Fernanda Coelho de Souza, Eric G. Cosio, Nallaret Davila Cardozo, Richarlly da Costa Silva, Mathias Disney, Javier Silva Espejo, Ted R. Feldpausch, Leandro Ferreira, Leandro Giacomin, Niro Higuchi, Marina Hirota, Euridice Honorio, Walter Huaraca Huasco, Simon Lewis, Gerardo Flores Llampazo, Yadvinder Malhi, Abel Monteagudo Mendoza, Paulo Morandi, Victor Chama Moscoso, Robert Muscarella, Deliane Penha, Mayda Cecília Rocha, Gleicy Rodrigues, Ademir R. Ruschel, Norma Salinas, Monique Schlickmann, Marcos Silveira, Joey Talbot, Rodolfo Vásquez, Laura Vedovato, Simone Aparecida Vieira, Oliver L. Phillips, Emanuel Gloor, David R. Galbraith

**Affiliations:** 1 School of Geography, University of Leeds, Leeds, UK; 2 Department of Ecology and Genetics, Evolutionary Biology Centre, Uppsala University, Uppsala, Sweden; 3 Department of Plant Biology, Institute of Biology, University of Campinas, Campinas, Brazil; 4 CREAF, Campus UAB, Cerdanyola del Vallés, Spain; 5 ICREA, Barcelona, Spain; 6 College of Life and Environmental Sciences, University of Exeter, Exeter, UK; 7 Department of Plant Biology, Institute of Biology, Programa de Pós Graduação em Biologia Vegetal, University of Campinas, Campinas, Brazil; 8 Institute of Systematic Botany and Ecology, Ulm University, Ulm, Germany; 9 Universidad Nacional de San Antonio Abad del Cusco, Cusco, Peru; 10 Programa de Pós-Graduação em Ecologia e Manejo de Recursos Naturais, Universidade Federal do Acre, Rio Branco, Brazil; 11 Department of Plant Biology, Institute of Biology, Programa de Pós Graduação em Ecologia, University of Campinas, Campinas, Brazil; 12 Departamento de Ciências Biológicas, Universidade do Estado de Mato Grosso (UNEMAT), Nova Xavantina, Brazil; 13 Programa de Pós-Graduação em Ecologia e Conservação, Universidade Federal do Paraná, Curitiba, Brazil; 14 Environmental Change Institute, School of Geography and the Environment, University of Oxford, Oxford, UK; 15 AMAP (Botanique et Modélisation de l’Architecture des Plantes et des Végétations), CIRAD, CNRS, INRA, IRD, Université de Montpellier, Montpellier, France; 16 School of Geography, University of Birmingham, Birmingham, UK; 17 Birmingham Institute of Forest Research (BIFoR), Birmingham, UK; 18 School of Geosciences, University of Edinburgh, Edinburgh, UK; 19 Research School of Biology, Australian National University, Canberra, Australian Capital Territory Australia; 20 Instituto de Geociências, Faculdade de Meteorologia, Universidade Federal do Pará, Belém, Brazil; 21 Pontificia Universidad Católica del Perú, Lima, Peru; 22 Universidad Nacional de la Amazonia Peruana, Iquitos, Peru; 23 Museo de Historia Natural Noel Kempff Mercado, Santa Cruz de la Sierra, Bolivia; 24 Universidad Autonoma Gabriel Rene Moreno, Santa Cruz, Bolivia; 25 Instituto de Biodiversidade e Florestas, Universidade Federal do Oeste do Pará, Santarém, Brazil; 26 Universidad Nacional de la Amazonia Peruana (UNAP), Iquitos, Peru; 27 Instituto de Investigaciones de la Amazonia Peruana, Iquitos, Peru; 28 National Institute for Space Research (INPE), São José dos Campos-SP, Brazil; 29 Universidade de Sāo Paulo (USP), São Paulo, Brazil; 30 Laboratório de Botânica e Ecologia Vegetal, Universidade Federal do Acre, Rio Branco, Brazil; 31 SOS Amazônia, Programa Governança e Proteção da Paisagem Verde na Amazônia, Rio Branco-AC, Brazil; 32 Universidade Federal do Acre, Rio Branco, Brazil; 33 Department of Forestry, University of Brasilia, Campus Darcy Ribeiro, Brasília, Brazil; 34 Sección Química, Pontificia Universidad Católica del Perú, Lima, Peru; 35 Instituto Federal de Educação, Ciência e Tecnologia do Acre, Campus Baixada do Sol, Rio Branco, Brazil; 36 Department of Geography, University College London, London, UK; 37 Departamento de Biología, Universidad de La Serena, La Serena, Chile; 38 Museu Paraense Emílio Goeldi, Belém, Brazil; 39 Departamento de Sistemática e Ecologia, Centro de Ciências Exatas e da Natureza, Universidade Federal da Paraíba, João Pessoa, Brazil; 40 Instituto Nacional de Pesquisas da Amazônia, Manaus, Brazil; 41 Department of Physics, Federal University of Santa Catarina, Florianópolis, Brazil; 42 Universidad Nacional Jorge Basadre de Grohmann (UNJBG), Tacna, Peru; 43 Jardín Botánico de Missouri, Oxapampa, Peru; 44 Programa de Pós-Graduação em Biodiversidade, Universidade Federal do Oeste do Pará, Santarém, Brazil; 45 Instituto de Ciências e Tecnologia das Águas, Universidade Federal do Oeste do Pará, Santarém, Brazil; 46 Programa de Pós-Graduação em Botânica, Instituto Nacional de Pesquisas da Amazônia, Manaus, Brazil; 47 Embrapa Amazonia Oriental, Belém, Brasil; 48 Museu Universitário, Centro de Ciências Biológicas e da Natureza, Universidade Federal do Acre, Rio Branco, Brazil; 49 Institute for Transport Studies, University of Leeds, Leeds, UK; 50 Núcleo de Estudos e Pesquisas Ambientais, Universidade Estadual de Campinas, Campinas, Brazil

**Keywords:** Ecophysiology, Forest ecology, Tropical ecology, Biodiversity

## Abstract

Tropical forests face increasing climate risk^1,2^, yet our ability to predict their response to climate change is limited by poor understanding of their resistance to water stress. Although xylem embolism resistance thresholds (for example, $\varPsi$_50_) and hydraulic safety margins (for example, HSM_50_) are important predictors of drought-induced mortality risk^3–5^, little is known about how these vary across Earth’s largest tropical forest. Here, we present a pan-Amazon, fully standardized hydraulic traits dataset and use it to assess regional variation in drought sensitivity and hydraulic trait ability to predict species distributions and long-term forest biomass accumulation. Parameters $\varPsi$_50_ and HSM_50_ vary markedly across the Amazon and are related to average long-term rainfall characteristics. Both $\varPsi$_50_ and HSM_50_ influence the biogeographical distribution of Amazon tree species. However, HSM_50_ was the only significant predictor of observed decadal-scale changes in forest biomass. Old-growth forests with wide HSM_50_ are gaining more biomass than are low HSM_50_ forests. We propose that this may be associated with a growth–mortality trade-off whereby trees in forests consisting of fast-growing species take greater hydraulic risks and face greater mortality risk. Moreover, in regions of more pronounced climatic change, we find evidence that forests are losing biomass, suggesting that species in these regions may be operating beyond their hydraulic limits. Continued climate change is likely to further reduce HSM_50_ in the Amazon^6,7^, with strong implications for the Amazon carbon sink.

## Abstract (web summary)

A pan-Amazon study of forests shows large variations in drought tolerance traits and finds that forests in regions of pronounced climate change are losing biomass and may be operating beyond their hydraulic limits.

## Main

Rising temperatures and drought pose a significant challenge to the functioning of Earth’s forests and may already be changing forest dynamics globally^8,9^. The consequences of intensifying climate stress may be particularly marked in Amazon rainforests, which house around 16,000 tree species^10^, store more than 100 Pg of carbon in their biomass^11^ and regulate climate through their substantial exchanges of carbon, water and energy with the atmosphere^12^. Recent recurrent drought events across the Amazon have increased tree mortality^13,14^ and may be partially responsible for the long-term decline of the Amazon carbon sink^15,16^. Water stress over Amazonian forests is likely to intensify under future climate due to increasing temperatures, altered rainfall and increased occurrence of extreme events^1,2^. Thus, understanding the vulnerability of these forests to drought stress is of great importance.

Substantial evidence points to hydraulic failure, defined as a disruption of whole-plant water transport capacity due to embolism of xylem vessels^17^, as a key mechanism underpinning drought-induced mortality^3,4,18^. The vulnerability of trees to hydraulic failure is closely related to their ability to resist xylem embolism and the proximity with which they operate to critical embolism thresholds, their hydraulic safety margins (HSMs)^4,5^. Commonly used metrics of embolism resistance include the xylem water potentials at which 50% ($\varPsi$_50_) and 88% ($\varPsi$_88_) of stem hydraulic conductance are lost, whereas HSMs integrate these embolism resistance thresholds with in situ atmospheric vapour pressure and soil water status, through several physiological and allometric traits^19,20^ and denote how close midday water potentials measured at the peak of the dry season ($\varPsi$_dry_) in the field approach $\varPsi$_50_ (HSM_50_) or $\varPsi$_88_ (HSM_88_)^4,18,21^. Thus, HSMs provide a combined measure of xylem vulnerability and exposure to water deficit. These properties have been shown to be important predictors of mortality under drought^22^ and are central to efforts to understand and mechanistically model climate change impacts on vegetation function^8,23–25^

Several recent studies have evaluated tree hydraulic properties within^3,26–33^ and between sites^6,34^ in the central and eastern Amazon. However, most of these sites share broadly similar climate, are located on highly weathered, infertile soils and are amongst the least dynamic Amazonian forests^35,36^. A basin-wide perspective of how hydraulic properties vary across Amazonian forests, which encompass a broad range of geographic/climatic conditions and species composition, is lacking at present, limiting understanding of how climate change will impact this critical ecosystem.

**Fig. 1:**
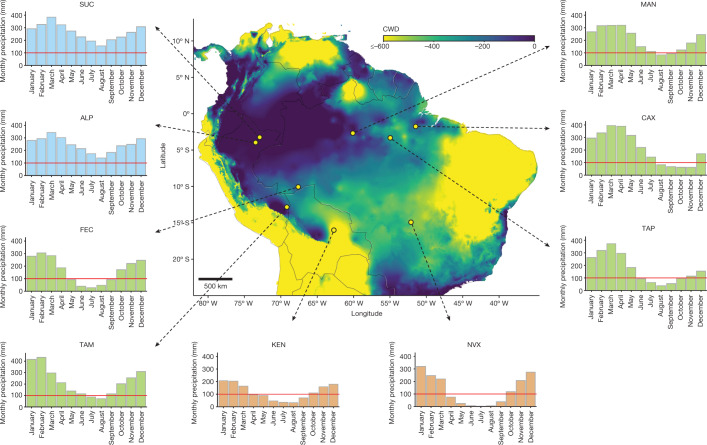
Sampled sites: spatial distribution and climatological variation. The map depicts long-term climatical water deficit (CWD) obtained from ref. ^63^ (2.5 arcsec resolution). Bar graphs show mean precipitation per month (1998–2016) per site. The red lines at 100 mm represent the definition of dry season, where the monthly precipitation is below 100 mm. Precipitation data were obtained from TRMM (the Tropical Rainfall Measuring Mission—TMPA/3B43 v.7) at 0.25^°^ spatial resolution^64^. Aseasonal ever-wet sites (blue bars): Sucusari (SUC) and Allpahuayo (ALP-1 and ALP-2). Intermediate DSL sites (green bars): Acre (FEC), Caxiuanã (CAX), Manaus (MAN), Tambopata (TAM) and Tapajós (TAP). Ecotonal long DSL sites (brown bars): Kenia (KEN-1 and KEN-2) and Nova Xavantina (NVX).

**Extended Data Fig. 1:**
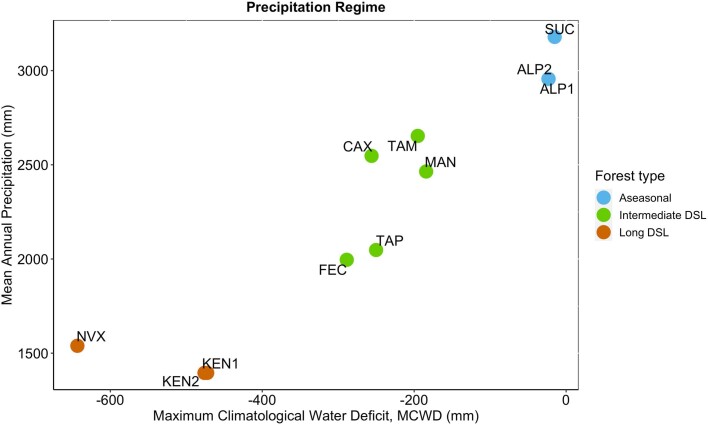
Precipitation regimes of sampled sites. Precipitation data were obtained from TRMM (the Tropical Rainfall Measuring Mission—TMPA/3B43 version 7)^64^ at 0.25^o^ spatial resolution from 1998–2016. Maximum cumulative water deficit (MCWD) was computed following Aragão et al. (2007) but replacing universal ET values with site-specific values derived from the ERA-5 re-analysis product^71^. MCWD is defined as the maximum climatologically-induced water deficit (see equation 1 in Methods). Sites in which MCWD~0 do not experience seasonality (dry season length (DSL) = 0), while sites with very negative MCWD values are strongly seasonally water-stressed. Sites are colour-coded by forest types, based on their seasonal rainfall patterns: aseasonal (blue), intermediate DSL (green) and long DSL (brown).

**Extended Data Fig. 2:**
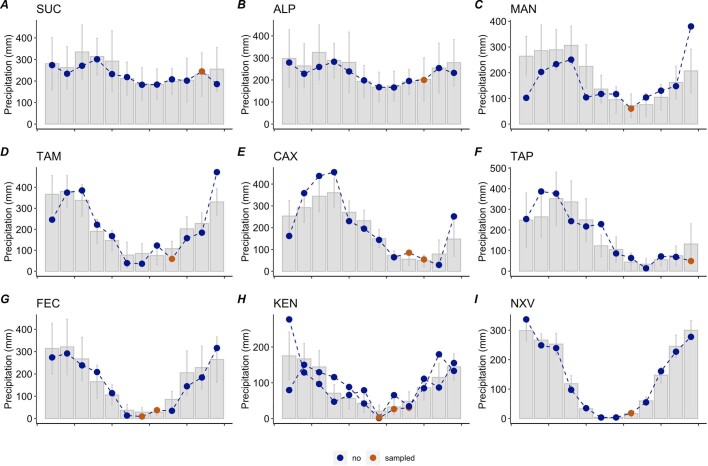
Climatological data corresponding to *Ψ*_dry_ (*in situ* dry season leaf water potential) sampling at each site. Grey bars and error bars show the mean and standard deviation of monthly precipitation from 1991 to 2018 (CRU data ts.4.0338)^75^. The blue dashed lines represent the year of sampling, while the brown points show the months at which $\varPsi$_dry_ was measured. Hydraulic traits and climatic data for TAP were obtained from Brum et al.^27^. We display CRU data in this figure due to no availability of TRMM (Tropical Rainfall Measuring Mission) data beyond 2016. Sampling years can be found in Supplementary Table 8.

**Extended Data Fig. 3:**
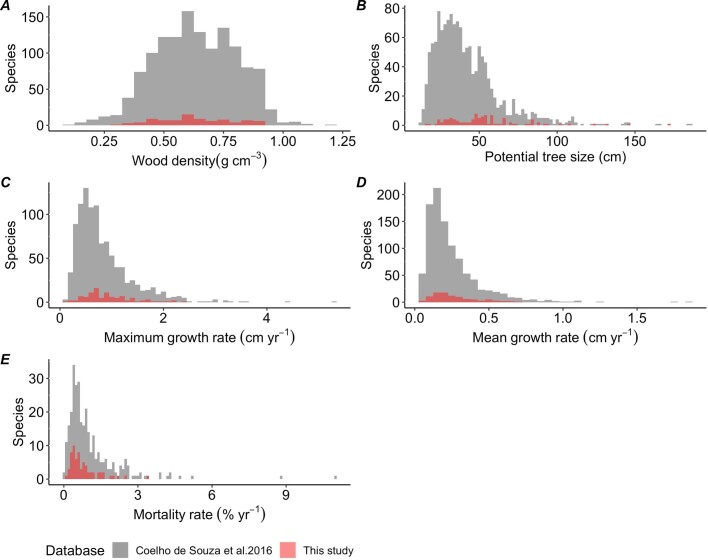
Functional trait range of species sampled in this study. Histograms of life-history related traits of the sampled species (red) in relation to comprehensive histograms of the broader Amazon tree flora (grey). A) Mean wood density (g cm^−3^)^65,66^; Potential size, calculated as the 95th percentile of diameter distribution (cm); C) Maximum growth, calculated as the 95th percentile of individual growth rates available for a given species (cm yr^−1^); D) Mean growth rate (cm yr^−1^); E) Mean mortality rate (% yr^−1^). All trait data shown in this figure were extracted from Coelho de Souza et al.^37^.

Here, we present a pan-Amazon dataset of plant hydraulic properties ($\varPsi$_50_, HSM_50_ and $\varPsi$_dry_), following a fully standardized methodology. Our dataset includes hydraulic traits (HTs) from 129 species across 11 forest plots in the eastern, central eastern and southern Amazon. Our sampling spans the entire Amazonian precipitation space ranging from ecotonal forests at the biome edges with long dry season length (DSL) to ever-wet aseasonal forests (Fig. 1, Extended Data Fig. 1 and Supplementary Table 1). In each site, sampling effort was concentrated on adult dominant canopy and subcanopy species (Supplementary Table 2). For each species at each site, we constructed xylem embolism vulnerability curves (describing the reduction in hydraulic conductivity with declining water potential), from which we determined $\varPsi$_50_ and $\varPsi$_88_ and also measured midday leaf water potential during the peak of the dry season ($\varPsi$_dry_; Extended Data Fig. 2) to compute hydraulic safety margins (HSM_50_ = $\,\varPsi$_dry_ − $\,\varPsi$_50_). Collectively, the species sampled encompass a wide array of life-history strategies^37^ and represent about 24% of total Amazon tree biomass, excluding palms^38^ (Extended Data Fig. 3).

We use this dataset to assess basin-wide biogeographic variation in embolism resistance and vulnerability to hydraulic failure. Finally, we take advantage of standardized long-term inventory plots distributed across the Amazon^39^, within which our sites are nested, to test whether these traits predict Amazonian species distribution and long-term aboveground biomass (AGB) accumulation (that is, the forest AGB carbon sink).

## Hydraulic traits distribution

**Fig. 2:**
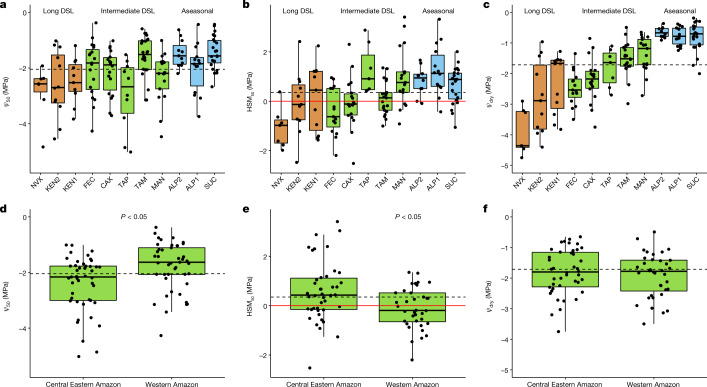
Hydraulic trait variation across and within Amazon forest sites. **a**,**d**, Xylem water potential at which 50% of the conductance is lost ($\varPsi$_50_). **b**,**e**, HSMs related to $\varPsi$_50_ (HSM_50_ = $\varPsi$_dry_ − $\varPsi$_50_). **c**,**f**, In situ dry season leaf water potential ($\varPsi$_dry_). **d**–**f**, Show hydraulic trait variation within intermediate DSL forests, subsetted according to Amazon region (central eastern Amazon: TAP, CAX and MAN; western Amazon: FEC and TAM). Dashed lines denote the mean value of each trait across all tree taxa in the dataset whereas the red line indicates HSMs equal to zero. Boxplots show the 25th percentile, median and 75th percentile. The vertical bars show the interquartile range ×1.5 and datapoints beyond these bars are outliers. Sites are sorted according to increasing water availability. Red, green and blue colours represent sites from ecotonal long DSL, intermediate DSL and aseasonal ever-wet forests, respectively. Each point represents one species per site (*N*_total_ = 170 species). Significant differences at *P* < 0.05 are shown on the figure (Wilcoxon rank sum tests).

**Extended Data Fig. 4:**
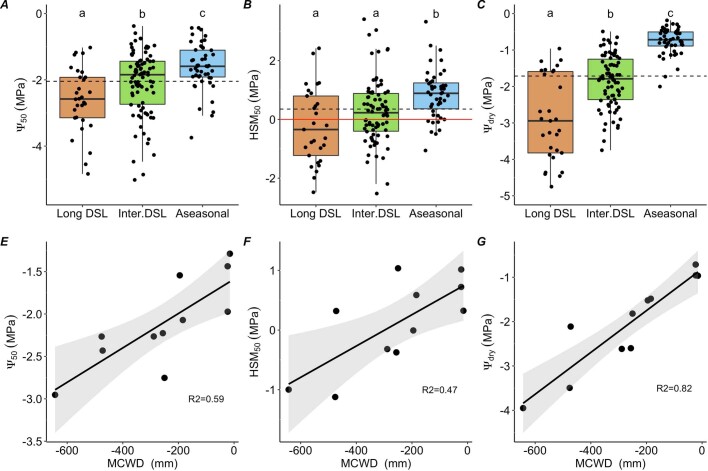
Hydraulic traits variation at species and community level. Top panels: variation in hydraulic traits across Amazon forest types; long DSL (brown), intermediate DSL (green) and aseasonal (blue). A) xylem water potential at which 50% ($\varPsi$_50_) of the conductance is lost. B) hydraulic safety margins related to $\varPsi$_50_ (HSM_50_ = $\varPsi$_dry_ – $\varPsi$_50_). C) minimum leaf water potential observed in the dry season ($\varPsi$_dry_). Dashed lines show the mean value of each trait across all tree taxa. Red lines denote hydraulic safety margins equal to zero. Significant differences at *p* < 0.05 are shown by letters above each boxplot (Kruskal–Wallis followed by post hoc Mann–Whitney–Wilcoxon Rank Sum test). Each point represents one species per site. Long DSL, intermediate DSL and aseasonal forests encompass 3, 5 and 3 forest sites, respectively. Boxplots display the 25^th^ percentile, median and 75^th^ percentile. The vertical bars show the interquartile range times 1.5 and datapoints beyond these bars are outliers. Bottom panels: Relationship between tree basal-area weighted mean hydraulic traits and maximum cumulative water deficit (MCWD). D) Basal area-weighted mean $\psi$_50_ (xylem water potential at which 50% of the conductance is lost); E) Basal area-weighted mean hydraulic safety margin (HSM_50_); F) Basal area-weighted mean minimum leaf water potential observed in the dry season ($\varPsi$_dry_); n = 11 sites. Significant linear relations are shown by regression lines and 95% confidence intervals, by shaded areas. See methods for MCWD calculations.

Our analyses suggest a strong overarching effect of water availability on HTs across Amazonian forests, both in terms of species level and community values. As expected, species found in ever-wet aseasonal forests (DSL of 0 months) have the least resistant xylem (least negative $\varPsi$_50_), whereas forests with intermediate DSL (2–5 months) and ecotonal long DSL forests (DSL of more than 5 months) have species with progressively more resistant (more negative $\varPsi$_50_) xylem tissue (*P* < 0.0001; Fig. 2 and Extended Data Fig. 4). The same pattern is observed for $\varPsi$_dry_, whereby species in long DSL forests experience more negative $\varPsi$_dry_ than those in intermediate DSL or ever-wet aseasonal forests (*P* < 0.0001; Fig. 2 and Extended Data Fig. 4). Contrary to the convergence in HSM_50_ reported by previous^21,40^ (but not all^41^) studies across woody species at continental and global scales, we find that HSM_50_ varies significantly across Amazonian forests (*P* < 0.0001; Fig. 2 and Extended Data Fig. 4). Species in ever-wet aseasonal forests generally have higher HSM_50_ than those in intermediate DSL and long DSL forests and thus face the lowest apparent risk of hydraulic failure despite having xylem that is least resistant to embolism. This may reflect a lack of exposure to drought in ever-wet forests. Similar patterns are also observed at the community level. Across all sites, basal area weighted HT are strongly related to maximum cumulative water deficit (MCWD; Extended Data Fig. 4), which alone explains 59%, 47% and 82% of the observed variation in $\varPsi$_50_, HSM_50_ and $\varPsi$_dry_ (linear model: *P* = 0.004, *P* = 0.01, *P* < 0.0001), respectively. Drier sites are generally more resistant to embolism but have lower HSM than do wetter sites, in agreement with recent global analysis^41^. Many species in the driest sites have negative HSM_50_ (Fig. 2b), suggesting that (1) they may be adapted to cope with seasonal exceedance of HSM_50_ and (2) mortality thresholds in these regions may be associated with higher conductance losses; for example, HSM_88_ as has been reported in experimental studies^5,42^_._ Although our results point to a very strong control of background climate (MCWD) in driving variation in hydraulic properties across the Amazon, we note that other factors governing water availability locally are also probably important, including topography-associated variation in water table depth^28,43^.

**Extended Data Fig. 5:**
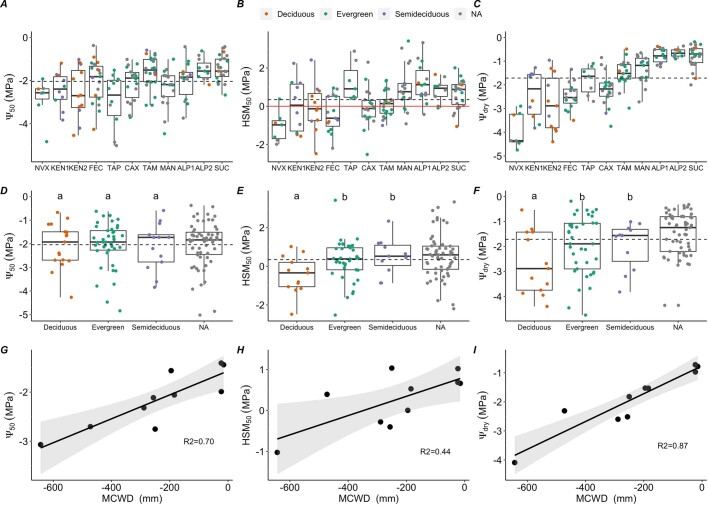
Leaf habit information of the sampled species per plot (top panels), hydraulic trait variation across leaf habit groups (middle panels) and relationship between basal area-weighted mean hydraulic traits and maximum cumulative water deficit when excluding deciduous and semideciduous species. A) and D) xylem water potential at which 50% of the conductance is lost ($\varPsi$_50_); B) and E) Hydraulic safety margins related to $\varPsi$_50_ (HSM_50_ = $\varPsi$_dry_ – $\varPsi$_50_ and C) and F) Minimum *in situ* leaf water potential observed in the dry season ($\varPsi$ dry). Dashed lines denote the mean value of each trait across all tree taxa in the dataset. Red line, the hydraulic safety margins equal to zero. Boxplots display the 25^th^ percentile, median and 75^th^ percentile. The vertical bars show the interquartile range times 1.5 and datapoints beyond these bars are outliers. Sites are sorted by increasing water availability. Each point represents one species per site (N = 170 species) in the top panels and species mean ($\varPsi$_50,_ HSM_50_) and species minimum $\varPsi$_dry_ per leaf habit in the bottom panels (N = 136 species). Deciduous, semideciduous and evergreen species are represented by red, blue and green points, respectively. Grey points or NA represent species for which leaf habit information was not available. Significant differences at *p* < 0.05 are represented by different letters above each boxplot (Kruskal–Wallis followed by post hoc Mann–Whitney–Wilcoxon Rank Sum test). G) $\psi$_50_ (xylem water potential on which 50% of the conductance is lost; H) Hydraulic safety margin (HSM_50_ = $\varPsi$_dry_ – $\varPsi$_50_); I) *In situ* leaf water potential observed in the dry season ($\varPsi$ dry); n = 10 sites. Significant linear relations are shown by regression lines. The shaded area represents the 95% confidence interval of the regression slope. Further leaf habit information of sampled species is provided in Supplementary Table 10.

The relationship between community mean HSM_50_ and MCWD is unlikely to be driven by differences in leaf phenology across sites. Within our dataset, we find that deciduous species have lower HSM_50_ than do semideciduous and evergreen species (Extended Data Fig. 5), consistent with other findings that deciduous species have hydraulically riskier strategies^44^. However, the relationship between HSM_50_ and MCWD remains, even when deciduous and semideciduous species are excluded from the analysis (*P* = 0.02, *R*^2^ = 0.44; Extended Data Fig. 5). Thus, deciduousness may partially explain the low HSM_50_ observed in the dry fringes of the Amazon but further explanations are required. In these regions, where climate change is most accentuated, trees may now be operating at their physiological limits.

Within intermediate DSL forests, despite relatively similar MCWD and annual rainfall, species in central eastern Amazon have more resistant xylem and have wider HSM_50_ than their generally more dynamic western Amazon counterparts (*P* = 0.001; Fig. 2). Indeed, whereas resistance to embolism of intermediate DSL forests in western Amazon (mean $\varPsi$_50_ = −1.77 ± 0.13 MPa) is similar to that of aseasonal forests (mean $\varPsi$_50_ = −1.61 ± 0.1 MPa), intermediate DSL forests in central eastern Amazon (mean $\varPsi$_50_ = −2.40 ± 0.15 MPa) have embolism resistance similar to ecotonal forests in southern Amazon (mean $\varPsi$_50_ = −2.59 ± 0.18 MPa). On the other hand, $\varPsi$_dry_ does not significantly differ between these forests (*P* = 0.5), indicating that western Amazon forest species do not compensate for their more vulnerable xylem through tighter leaf water potential regulation . Rather, western Amazon species show markedly lower HSM_50_ (mean HSM_50_ = −0.07 ± 0.14 MPa) than do central eastern species occupying a similar climatic niche (mean HSM_50_ = 0.58 ± 0.19 MPa, *P* = 0.01).

## HTs explain Amazon tree biogeography

**Fig. 3:**
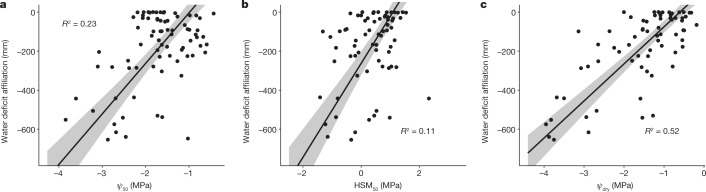
Relationship between WDA and HTs across western Amazon tree species. **a**–**c**, Embolism resistance $\varPsi$_50_ (**a**), hydraulic safety margin HSM_50_ (**b**) and minimum leaf water potential observed in the dry season $\varPsi$_dry_ (**c**). Individual points indicate species mean trait values (*n* = 87). Less negative WDA values denote wet-affiliated species and more negative WDA denote dry-affiliated species. Species-level WDA data were obtained from ref. ^45^. SMA regressions are shown by solid lines. The grey shaded areas represent the 95% bootstrapped confidence intervals for the slopes and intercepts. The *R*^2^ of each regression is shown on the figure. For this analysis, we subset our dataset to include only species collected in the western Amazon as done by ref. ^45^.

It has been shown previously that the distribution of tree species in western Amazon is strongly modulated by water availability, with some species associated with wet environments and others with dry^45^. We find a positive relationship between all evaluated HTs and species water deficit affiliation (WDA)^45^, defined as species preference for wet or dry habitat on the basis of its relative abundance across the precipitation space over which it is found (Fig. 3). Taxa with more negative WDA (dry-affiliated taxa) are widely spread in the Neotropics^45^. Although dry-affiliated taxa can in principle also occur in wet places, this is not true for most Amazonian species, which are highly wet-affiliated and not found in drier environments^45^. As expected, we find a significant positive relationship (*R*^2^ = 0.52, *P* *<* 0.0001) between $\varPsi$_dry_ and WDA (Fig. 3); that is, species associated with drier bioclimates experience more negative water potentials. A significant relationship between $\varPsi$_50_ and WDA (*R*^2^ = 0.23, *P* *<* 0.0001) further reveals that the xylem of species found in drier climates is more adapted to deal with lower water potentials than that of wet-affiliated species. These findings are qualitatively consistent with a worldwide study showing that conifer species occurring in drier climates have xylem that is more resistant to embolism than those found in more mesic climates^46^. However, we still find a weak positive relationship between HSM_50_ and WDA (*R*^2^ = 0.11, *P* = 0.005), such that dry-affiliated species have lower HSM_50_ than do wet-affiliated species and thus face greater hydraulic risk (Fig. 3). Continuation of drying trends observed in the southern Amazon^47^ will probably further reduce $\varPsi$_dry_ and HSM_50_ of tree species found in this region, assuming limiting acclimation in $\varPsi$_50_, as documented by other authors (for example, ref. ^30^).

## HSMs predict Amazonian carbon balance

**Fig. 4:**
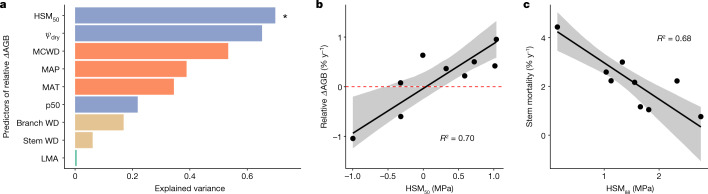
Relationship between relative *Δ*AGB and basal area weighted mean vegetation traits and climatic factors across clusters of the Amazonian forests. **a**, Variance explained by individual predictors when using SMA models to predict plot-level relative *Δ*AGB, with *Δ*AGB calculated as (AGB_end_ − AGB_start_)/period of monitoring length/standing woody biomass. Climatic data (MAT, MAP and MCWD), HTs ($\varPsi$_50_, $\varPsi$_dry_ and HSM_50_, defined as the difference between $\varPsi$_dry_ and $\varPsi$_50_) and other plant traits (stem and branch wood density and LMA) are indicated as red, blue, brown and green bars, respectively. Asterisk denotes statistically significant bivariate relationships after correcting for multiple hypothesis testing, using Bonferroni-corrected *P* < 0.05. Stem wood density values were extracted from the Global Wood Density database^65,66^. Bivariate plots and statistics for all predictor variables considered are shown in Extended Data Fig. 6 and Supplementary Table 3. **b**, Relationship between basal area weighted mean HSM_50_ and plot-level relative *Δ*AGB. We computed relative *Δ*AGB due to high standing AGB variance across plots. However, we also repeated B regression by considering absolute *Δ*AGB and this result was independent of whether absolute or relative *Δ*AGB were used in the bivariate regressions (Extended Data Fig. 7d). **c**, Relationship between basal area weighted mean HSM_88_ and annual instantaneous stem mortality rate (equation (4); ref. ^67^) across forest plots. The solid line is the best fit line of the SMA model and the shaded area represents the 95% bootstrapped confidence interval. KEN plots were excluded from all forest dynamics analyses because of a fire event that occurred in the region in 2004^68^ and may still be affecting biomass accrual.

**Extended Data Fig. 6:**
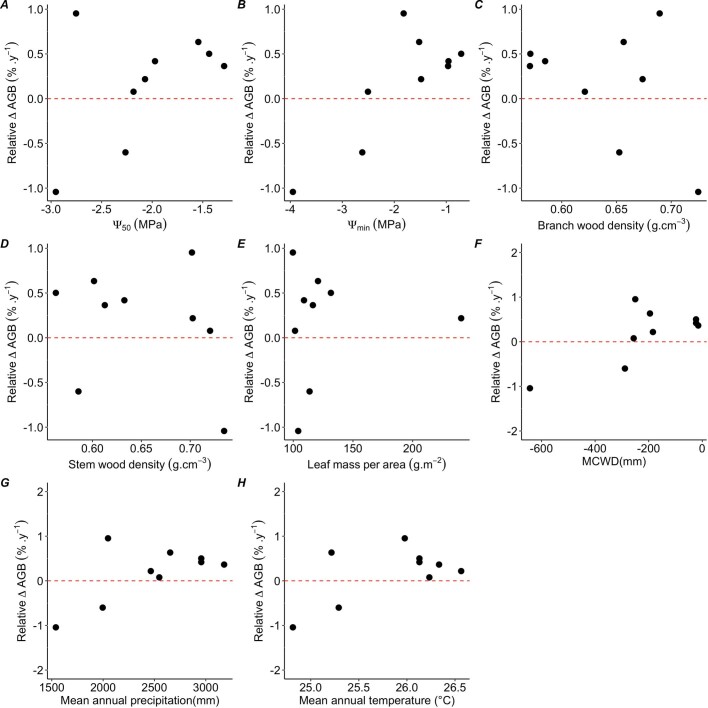
Bivariate relationship between plot relative aboveground net biomass change (ΔAGB) and basal-area-weighted mean vegetation traits (A–E) and climatic variables (F–I) across Amazonian forest plots. Due to high standing aboveground biomass variability across sites, we computed relative ΔAGB values as: ((AGB _end_ – AGB _start_)/census length)/AGB (time-weighted mean standing woody biomass). Each point indicates a forest plot. Information about each plot and the observation period used can be found in Supplementary Table 5. Stem wood density data were extracted from the Global Wood Density database^65,66^. KEN plots were excluded from all forest dynamics analyses because of a fire event that occurred in the region in 2004^66^ and may still be affecting biomass accrual. Regression lines show significant relationships using standard major axis (SMA) models after Bonferroni correction for multiples hypothesis testing. Supplementary Table 3 shows the results of the SMA models.

**Extended Data Fig. 7:**
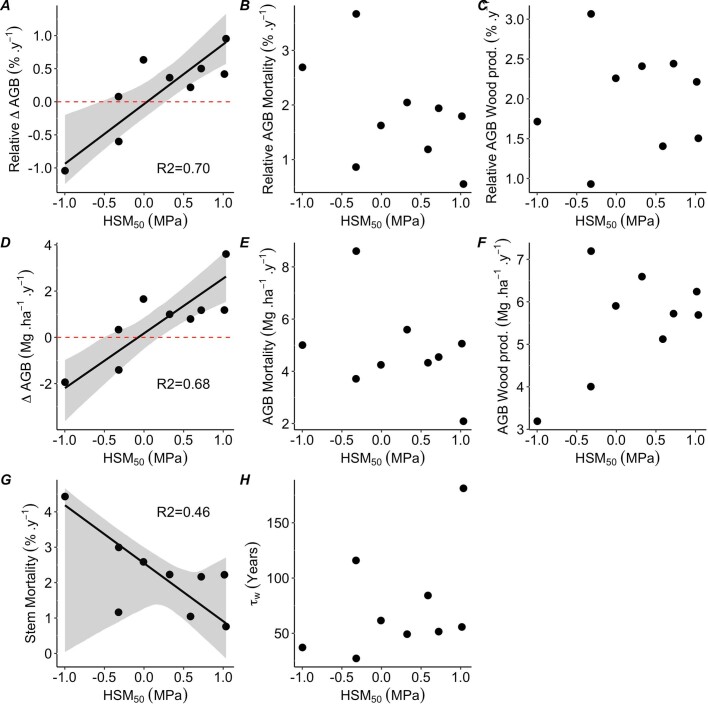
Plot-level analyses: bivariate relationships between basal area-weighted mean hydraulic safety margin and forest dynamics across Amazonian forest plots. Basal area-weighted mean HSM_50_ in relation to relative (A-C) and absolute (D-H) forest dynamics values. A) Relative annual aboveground biomass net change (ΔAGB/AGB), where: ΔAGB is the difference in aboveground biomass between the final and initial censuses (AGB_final census_ – AGB_initial census_) divided by total monitoring length for that plot (Date_final census_ – Date_initial census_) in years and AGB is the time-weighted mean standing woody biomass across censuses per plot; B) Relative annual mortality in terms of biomass: (AGB_MORT_/AGB). Where AGB_MORT_ is the sum of the AGB of all dead trees and the unobserved components (see methods), divided by the census interval length^95^;C) Relative annual AGB wood productivity: (AGWP/AGB), where AGWP is defined as the sum of the biomass growth of surviving trees >10 cm DBH, new recruits > 10 cm DBH and the unobserved components (see methods), within a plot in a given census interval, divided by the census interval length^95^; D) Annual aboveground biomass net change (ΔAGB); E) Annual aboveground biomass mortality (AGB_MORT_); F) Annual aboveground wood productivity (AGWP), G) Annual instantaneous stem mortality rate (See methods Equation 4)^67^; H) Residence time of woody biomass calculated as the ratio of mean standing biomass to mean biomass mortality rate^52^. All these parameters were calculated for each census interval and we calculated time-weighted mean to have one value per plot. Each point indicates one forest plot. Significant linear relations are shown by regression lines (Standard major axis models). The shade area represents the 95% bootstrapped confidence interval. Information about each plot and the observation period is available on Supplementary Table 5. Supplementary Table 4 shows the results of the SMA models.

**Extended Data Fig. 8:**
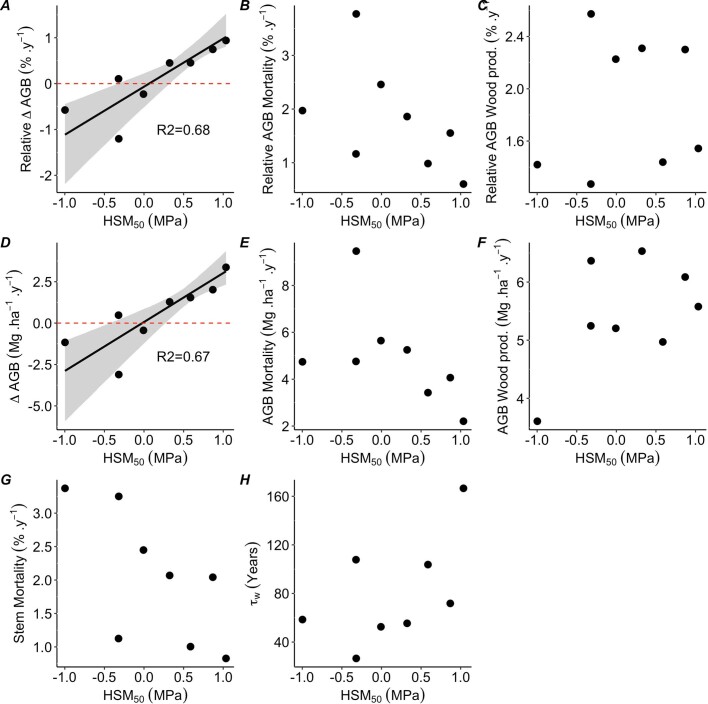
Cluster-level analyses: Relationship between basal area weighted mean HSM_50_ and cluster mean forest dynamics across clusters of forest plots. Basal area-weighted mean HSM_50_ in relation to cluster mean relative (A–C) and absolute (D–H) forest dynamics values. A) Relative annual aboveground biomass net change (ΔAGB/AGB), where: ΔAGB is the difference in aboveground biomass between the final and initial censuses (AGB_final census_ – AGB_initial census_) divided by total monitoring length for that plot (Date_final census_ – Date_initial census_) in years and AGB is the time-weighted mean standing woody biomass across censuses per plot; B) Relative annual mortality in terms of biomass: (AGB_MORT_/AGB). Where AGB_MORT_ is the sum of the AGB of all dead trees and the unobserved components (see methods), divided by the census interval length^95^;C) Relative annual AGB wood productivity: (AGWP/AGB), where AGWP is defined as the sum of the biomass growth of surviving trees >10 cm DBH, new recruits > 10 cm DBH and the unobserved components (see methods), within a plot in a given census interval, divided by the census interval length^95^; D) Annual aboveground biomass net change (ΔAGB); E) Annual aboveground biomass mortality (AGB_MORT_); F) Annual aboveground wood productivity (AGWP), G) Annual instantaneous stem mortality rate (see methods)^67^; H) Residence time of woody biomass calculated as the ratio of mean standing biomass to mean biomass mortality rate^52^. All these parameters were calculated for each census interval and we calculated time-weighted mean to have one value per plot. Each point indicates the mean value across clusters of forest plots, which in total encompass 31.37 ha of forest spread across 34 plots. Information about each cluster and the observation period used for each cluster is provided in Supplementary Table 5. The solid line is the best fit line of the standard major axis (SMA) model and the shaded area represents the 95% bootstrapped confidence interval. Supplementary Table 4 shows the results of the SMA models.

Forests across the Amazon have been gaining biomass in recent decades and this substantial carbon sink is estimated to account for 10–15% of the terrestrial land sink^15,48^. Forest inventory plots spread across the Amazon have revealed that Amazon forests vary widely in their biomass accumulation rates (*Δ*AGB, the difference between biomass gained by productivity and that lost by mortality) but the underlying mechanisms governing variation in *Δ*AGB across forests remain elusive^15,16^. We tested the predictive power of basal area weighted mean values of a range of plant traits including stem and branch wood density (WD_stem_ and WD_branch_), leaf mass per area (LMA) and HTs ($\varPsi$_50_, HSM_50_ and $\varPsi$_dry_), as well as climate metrics (for example, MCWD, mean annual precipitation (MAP) and mean annual temperature (MAT)) and found HSM_50_ to be the only significant predictor of the long-term aboveground net biomass change (*Δ*AGB) across forest plots (Fig. 4, Extended Data Fig. 6 and Supplementary Table 3). Although we cannot rule out the role of predictors for which we had no data (for example, root traits or pathogen status), this result highlights a key role for HSM_50_ in regulating forest dynamics (Extended Data Fig. 7 and Supplementary Table 4) and holds true when the analysis is repeated using dynamics data from a larger set of plots (clusters) located within the same landscape as the plots sampled directly for HTs (Extended Data Fig. 8 and Supplementary Tables 5 and  6).

**Extended Data Fig. 9:**
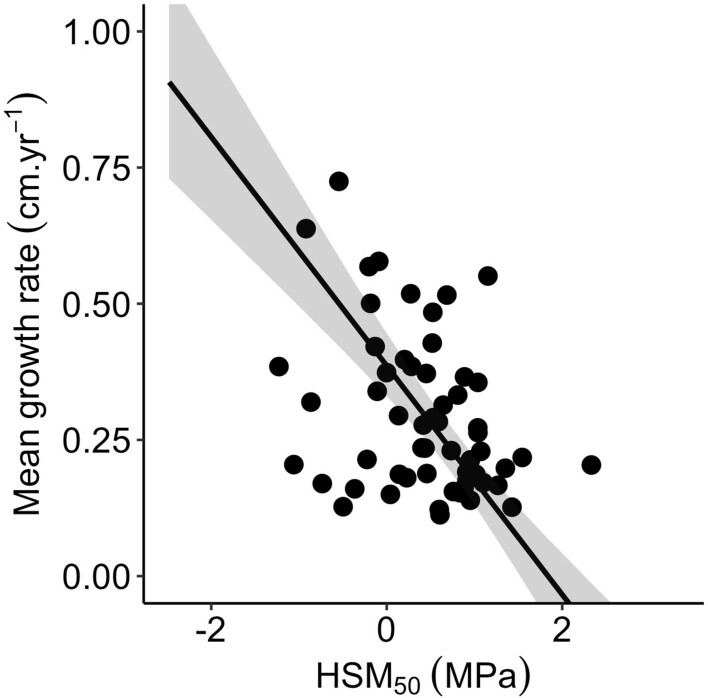
Relationship between published pan-Amazonian species-level growth rates^37^ and hydraulic safety margins. We restrict our analysis to the western Amazon, where there is the highest overlap of species between ours and Coelho de Souza et al.^37^ dataset and to avoid biases due to soil/forest dynamic differences across Amazonian regions. Hydraulic safety margins for species occurring across multiple sites were averaged to yield one value per species.

**Extended Data Fig. 10:**
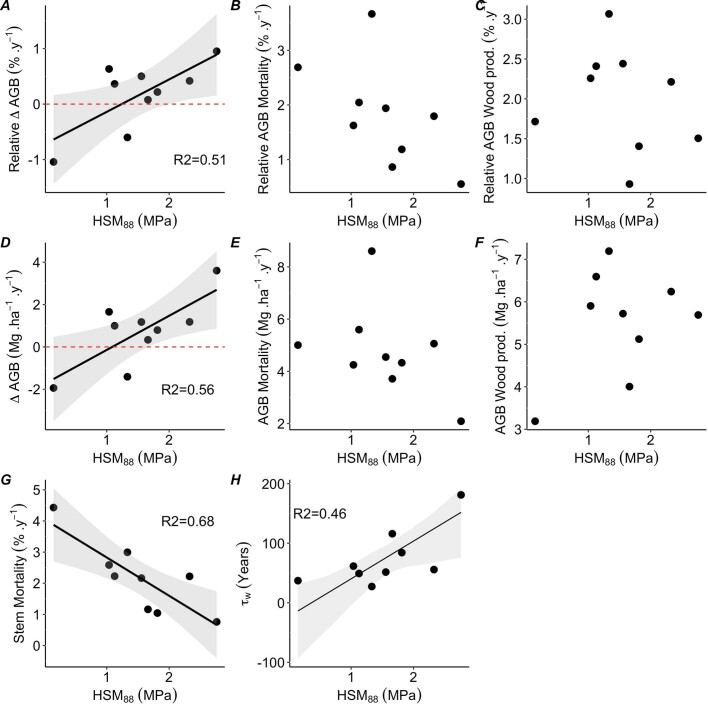
HSM_5_Plot-level analyses: bivariate relationships between basal area-weighted mean HSM_88_ and forest dynamics across Amazonian forest plots. Basal area-weighted mean HSM_88_ in relation to relative (A-C) and absolute (D-H) forest dynamics values. A) Relative annual aboveground biomass net change (ΔAGB/AGB), where: ΔAGB is the difference in aboveground biomass between the final and initial censuses (AGB_final census_ – AGB_initial census_) divided by total monitoring length for that plot (Date_final census_ – Date_initial census_) in years and AGB is the time-weighted mean standing woody biomass across censuses per plot; B) Relative annual mortality in terms of biomass: (AGB_MORT_/AGB). Where AGB_MORT_ is the sum of the AGB of all dead trees and the unobserved components (see methods), divided by the census interval length^95^;C) Relative annual AGB wood productivity: (AGWP/AGB), where AGWP is defined as the sum of the biomass growth of surviving trees >10 cm DBH, new recruits > 10 cm DBH and the unobserved components (see methods), within a plot in a given census interval, divided by the census interval length^95^; D) Annual aboveground biomass net change (ΔAGB); E) Annual aboveground biomass mortality (AGB_MORT_); F) Annual aboveground wood productivity (AGWP), G) Annual instantaneous stem mortality rate (see methods Equation 4)^67^; H) Residence time of woody biomass calculated as the ratio of mean standing biomass to mean biomass mortality rate^52^. All these parameters were calculated for each census interval and we calculated time-weighted mean to have one value per plot. Each point indicates one forest plot. Significant linear relations are shown by regression lines (Standard major axis models). The shade area represents the 95% bootstrapped confidence interval. Information about each plot and the observation period is available on Supplementary Table 5. Supplementary Table 4 shows the results of the SMA models.

HSM_50_ explained 70% of the variance in relative *Δ*AGB across Amazon forest plots and 67% of the absolute *Δ*AGB (*P* < 0.01 and *P* < 0.01, respectively, Extended Data Fig. 7 and Supplementary Table 4). Tree communities characterized by narrow HSM_50_ are gaining less biomass than those with high HSM_50_. Unravelling the physiological mechanisms underpinning the relationship between HSM_50_ and *Δ*AGB is challenging. The *Δ*AGB depends on the balance of productivity and mortality and HSM_50_ might be expected to affect both of these pathways. Uptake of CO_2_ for photosynthetic assimilation and transpirational water loss from leaves are directly coupled through stomata. The economic challenge of guaranteeing carbon gain although minimizing water loss gives rise to a range of plant strategies depending on resource availability^49^, with plants with acquisitive characteristics at one end of the spectrum to those with conservative characteristics at the other. Previous studies have shown that species with higher growth rates^50^ or with acquisitive trait attributes^51^ have lower HSMs. Using species-level diameter growth data from across the Amazon^37^, we also find a negative relationship with HSM_50_ (Extended Data Fig. 9). At the community scale, we generally find a stronger association of HSMs with mortality processes than with productivity, suggesting that HSM controls on mortality may be particularly important in regulating stand-level carbon balance. For example, both plot-level and cluster-level analyses show tighter relationships between HSM_50_ and relative AGB mortality (*R*^2^ of 0.26 and 0.27) than with relative AGB productivity (*R*^2^ of 0.00 and 0.02) (Extended Data Figs. 7 and 8), with the same patterns observed when HSM_88_ is considered instead of HSM_50_ (Extended Data Fig. 10 and Supplementary Table 4). We also find strong relationships between HSMs (HSM_88_ in particular) and woody biomass residence time (Extended Data Fig. 10). Relationships between HSM_50_ and stand-level stem mortality rates are invariably stronger than with biomass mortality metrics (plot level *R*^2^ = 0.47, *P* = 0.04; cluster level *R*^2^ = 0.47, *P* = 0.06) and are even stronger for HSM_88_, which was found to explain 68% of the variation in mortality rates at plot level (Fig. 4c;*R*^2^ = 0.68, *P* < 0.01), with similar patterns observed in the cluster-level analysis. These results indicate that exceedance of HSM_88_ greatly increases mortality risk and is consistent with experimental findings on saplings^42^.

We propose that the relationship between HSM and *Δ*AGB (Extended Data Fig. 7a,d) may be mediated mainly through HSM controls on woody biomass residence time (*τ*_w_), which in turn modulates forest response to a CO_2_ stimulus. Forests with high *τ*_w_ are expected to sustain CO_2_-induced net carbon gains for a longer period of time than forests with shorter *τ*_w_ as the lag times between productivity increases and knock-on increases in mortality are longer in high *τ*_w_ forests^52,53^. We find that forests with low HSM_50_/HSM_88_ tend to be associated with higher woody biomass turnover rates/lower woody biomass residence times (*τ*_w_; Extended Data Figs. 7h, 8h and 10h) but are often more productive than high HSM forests. In line with theoretical expectations, high *τ*_w_ forests have been found to be losing less biomass in the Amazon than those with low *τ*_w_ (ref. ^16^). High HSM_50_ may promote higher *τ*_w_ by reducing the risk of exceeding critical embolism resistance thresholds associated with tree mortality.

It has recently been proposed that HSM may also help to explain the growth–survivorship trade-off which is manifested at plot and at species level across the Amazon, whereby forests characterized by species with acquisitive traits that prioritize growth take greater hydraulic risks (that is, operate at lower HSM) and are more prone to mortality during periods of moderate water stress^54^. Our results support this as we find that species with high growth rates have low HSM_50_ (Extended Data Fig. 9), providing a potential mechanistic explanation for recent findings that high species-level growth rates are the principal mortality predictor for trees across the Amazon^55^. This HSM-mediated growth–survivorship trade-off also provides an explanation for why forests on more fertile, western Amazon forests have higher mortality rates than those in slower, less fertile central eastern Amazon forests^36,55^ as we find lower HSM in western Amazon forests occurring in a similar rainfall space to those in central eastern Amazon.

HSM_50_ reflects exposure to drought stress as well as plant water use strategies. Intensifying climate stress may help to explain why plots with the most negative HSM are losing rather than gaining biomass. The most vulnerable site (lowest HSM) in our study is in the southern fringe of the Amazon, the driest region of the Amazon and also the one that has also faced the greatest recent climatic changes^2,12^. The very low HSM_50_ observed there points to substantial hydraulic stress and may indicate that this region of the Amazon faces the most imminent climate risk. Our finding that forests in this region are losing biomass (Fig. 4b), is consistent with recent results based on analysis of atmospheric CO_2_ profiles that suggest remaining forests in the south eastern Amazon no longer act as a large-scale carbon sink^56^.

## Implications and conclusions

Our study evaluates large-scale variation in plant hydraulic properties across the Amazon. Our results provide compelling evidence for the importance of these properties in influencing basin-scale forest composition and function and offer important new insights into which Amazonian forests face greatest risk of drought-induced mortality. Although more resistant xylem (more negative $\varPsi$_50_) may provide Amazon species with an evolutionary adaptation to persist in water-limited environments, our results indicate that HSM_50_ is a powerful integrative trait that is strongly related to long-term ecosystem-scale biomass trajectories. We find that climatic factors alone or other plant traits do not have this explanatory power, in line with previous work suggesting that community-level variability in HSM_50_ exerts a strong control on ecosystem resilience to drought^57^. Although there are inevitable uncertainties (for example, precise determination of minimum water potential requires continuous measurements^58^ and other portions of the tree hydraulic pathway may show different sensitivities to water stress^59,60^), the fully standardized dataset allows direct comparison of the drought vulnerability of forests across the Amazon. We find that central eastern forests that have informed most of our current understanding of Amazon drought impacts are the least vulnerable to drought, possibly due to the periodic occurrences of El Niño/Southern Oscillation events and high climate variability creating a selection pressure for more drought-adapted taxa^61,62^. Of all sites considered in this study, the Tapajós site located close to one of the Amazon ecosystem-scale drought experiments has the most resistant $\varPsi$_50_ and the most positive HSM_50_, suggesting that upscaling of drought sensitivity inferred from these forests to the whole biome may underestimate Amazonian sensitivity to climate change. Continued increases in temperature and vapour pressure deficit, as predicted by all climate models, will probably reduce safety margins across Amazonian forests^6,31^ and further threaten the already declining Amazon carbon sink^15,56^. Our results indicate that these effects will be most marked in fast-turnover forests in western Amazon and increasingly stressed forests in the southern Amazon, which may already be at their physiological limit.

## Methods

### Site description

We assemble a pan-Amazon dataset of key HTs ($\varPsi$_50_, HSM_50_ and $\varPsi$_dry_), including 129 species distributed across 11 forest sites (Fig. 1 and Extended Data Fig. 1). The sites are old-growth lowland forests (less than 400 m of elevation), with no evidence of significant human disturbance, located in western, central eastern and southern Amazon. They were specifically chosen to span the full Amazonian precipitation gradient and to encompass the principal axes of species composition in the Amazon. The MAP varied from around 1,390 to around 3,170 mm yr^−1^ and mean MCWD varied from −640 to −15 mm across sites. Summary information for all sites can be found in Supplementary Tables 1 and 2.

### Species selection

To characterize drought sensitivity across a wide set of species and strategies, we sampled the most dominant adult canopy and subcanopy tree species at each site. For TAP, MAN and CAX, we used published data from refs. ^6,27,30^ which follow the same methodology as this study. The sampling effort at each site varied from 7 to 26 species which represented between 14% and 70% of the total basal area (Supplementary Table 2). Sites for which less than 30% of the total basal area was sampled (ALP-1, ALP-2, SUC, CAX and MAN) are hyperdiverse forests and lack the clear dominance structure by a few species observed in less diverse plots (for example, in the southern Amazon NVX site, the seven species sampled account for more than 50% of the basal area). Previous work by ref. ^6^, show that the MAN site, despite having the lowest sampled basal area of all sites presented in this study (about 14%) is representative of the broader floristic community, as adding a broader array of species-level hydraulic trait data did not significantly change basal area weighted mean (CWM) values. The same study found that mean species values are not likely to differ from community mean values if (1) species dominance is not driven by a few species, (2) traits have low dispersion around the mean (low standard deviation compared to the mean) and (3) traits are randomly distributed across species dominance distributions. For the other four sites for which sampled coverage was less than 30%, these criteria are generally satisfied (for example, cumulative dominance of the five most dominant species at ALP-1 is 27.9%, ALP-1 26.2%, SUC 15.0% and CAX 10.7%, standard deviation of $\varPsi$_50_ is between 32% and 49% of the mean value at each site and there is no relationship between species dominance and HT. Thus, basal area weighted mean trait values for the 11 sites probably well represent the broader unsampled community of trees.

### Abiotic data

To characterize climatological water deficit at each site, we calculated the MCWD^69^, which is a widely used measure of dry season intensity for Amazon forests^13,16,70^ that expresses the cumulative water stress experienced within an average year^69^. The MCWD metric assumes that a forest experiences water deficit if monthly precipitation does not meet evapotranspirational requirements and accumulates that deficit over all successive months with rainfall lower than evapotranspiration (*E*) values^69^. Monthly water deficit (WD_*n*_) was then calculated as the difference between precipitation (*P*) and evapotranspiration demand in each month *n*. MCWD was computed as the maximum monthly cumulative water deficit (CWD) experienced over an average year, for which the change in water deficit in any given month *n* is calculated as the difference between precipitation falling that month (*P*_*n*_) and an assumed evapotranspiration demand (*E*_*n*_, mm month^−1^). For any given month *n*,

1 $$\begin{array}{l}{{{\rm{CWD}}}_{n}={\rm{CWD}}}_{n-1}+{P}_{n}-{E}_{n}{\rm{;}}{\rm{\max }}({{\rm{CWD}}}_{n})=0{\rm{;}}\\ {{\rm{CWD}}}_{n}{\rm{MCWD}}={\rm{\min }}({\rm{CWD}}1,{\rm{CWD}}2,\ldots ,{\rm{CWD}}12)\end{array}$$

As all of our plots are in the southern hemisphere, their hydrological year coincides with the calendar year, allowing us to start our MCWD calculations at the beginning of each calendar year. For statistical analyses, we use the long-term mean MCWD for each location. Monthly precipitation data were obtained from the tropical rainfall measuring mission (TRMM TMPA/3B43 v.7)^64^ at 0.25° spatial resolution from 1998 to 2016. To estimate evapotranspiration, we used monthly ERA-5-Land Reanalysis E data at 0.1° spatial resolution from 1998 to 2016^71^, as this product has been suggested to well represent evapotranspiration estimates in the Amazon^72^. To have one value of evapotranspiration demand per site (*E*_*n*_ in equation (1)), we used the mean *E* value for the 3 months with highest *E* across years. Mean annual temperature data at 1 km spatial resolution were obtained from Worldclim2 (ref. ^73^).

We performed an alternative assessment computing MCWD on the basis of MOD16 (ref. ^74^) evapotranspiration product and on *E* estimation of 100 mm per month^69^ and we also computed MAP on the basis of TRMM^64^ and CRU^75^ data. The main results remained similar, independent of the climate product used (Supplementary Table 7).

### Collection of plant material

One fully sun-exposed top-canopy branch (or branch at the maximum height reachable by climbers) was collected from, on average, three individuals of each species at each site for subsequent construction of xylem vulnerability curves. The same or a second set of branches, in the same canopy position, was used to extract samples of wood density and LMA. For embolism resistance determination, data collection was undertaken during the wet season, when forests were maximally hydrated. Branches (more than 1 m long) were harvested during predawn or very early in the morning, to capture a fully hydrated starting point. Immediately after collection, basal portions of branches were wrapped with a wet cloth and branches were placed in a humidified opaque plastic bag to avoid desiccation during transport. Bags were sealed and carried to the field station for determination of xylem vulnerability curves. For samples not collected during predawn, branches were placed in a bucket, recut under water, covered with an opaque plastic bag and left to rehydrate for at least 5 h.

### Xylem embolism resistance ($\varPsi$_50_ and $\varPsi$_88_)

To quantify xylem resistance to embolism of Amazonian trees species, we focused on the water potentials associated with $\varPsi$_50_, given its wide use as a critical embolism resistance threshold^4,5^. To derive this parameter, we constructed xylem vulnerability curves by simultaneously measuring percentage of embolism formation and xylem water potential under progressive desiccation^76^. We estimated embolism using the pneumatic method of ref. ^77^, which quantifies the air extracted from within branches at each stage of dehydration and expresses this as a percentage of air discharge (PAD), defined as the percentage difference between the maximum amount of air removed under extreme dehydration (100% PAD) and the minimum amount removed under maximum hydration (0% PAD)^78^. For our measurements we used manual, self-constructed pneumatic devices, following ref. ^78^. Although automated devices for measuring air discharge are now available, these were not available at the time of our data collection. For all air discharge determinations, we applied the protocol of ref. ^77^ whereby measurements of air discharge were made over a 2.5 min interval. We note that the absolute volumes of air discharged are sensitive to the time interval of the discharge measurements, as shown by ref. ^79^,who report a difference of about 10% on the absolute air discharge measured for 15 s versus 115 s. There are still methodological uncertainties that require further investigation, including how the contribution of extraxylary discharge varies across different Amazonian species. Recent work using a pipe pneumatic model to simulate gas diffusion from intact conduits suggests that the overriding source of discharged air is from embolized xylem vessels although there is a small contribution (estimated to be about 9% over 15 s of discharge) from extraxylary pathways^80^. It is also important to note that the method measures embolism from vessels connected to the cut end of the branch from which gas is sampled and that there may be more embolism from vessels that are not directly connected to the cut end^80^. However, embolism spread during the branch dehydration method for embolism induction used in this study is expected to be predominantly from the cut branches^76^ and is corroborated by the strong agreement between petiole embolism status using the pneumatic method and leaf vein embolism assessed using optical approaches^81^.

The portability, ease of use and low cost of the pneumatic method make it ideally suited for use in remote tropical environments in which laboratory infrastructure is often minimal. Several studies have shown that $\varPsi$_50_ values derived from the pneumatic approach agree closely with those derived using more laborious methods^77,79,81–84^. For the TAP site in this study, $\varPsi$_50_ determinations based on the pneumatic method were compared with values derived from xylem vulnerability curves of percentage loss of conductance (PLC) constructed using a hydraulic ultralow flow meter^85^ and found a strong agreement (*R*^2^ = 0.83) between both methods^84^, further corroborating findings from previous studies (refs. ^27,84^ provide detailed description of the hydraulic method used). Although one study^86^ (but see refs. ^84,87^) proposed that the method may be unsuitable for long-vesseled species, we find no evidence of any vessel length bias in our $\varPsi$_50_ estimates derived from the pneumatic method (standard major axis (SMA) regression $\varPsi$_50_ versus maximum vessel length: *P* = 0.15, *R*^2^ = 0.02).

The initial PAD measurement for each branch was made immediately after removing the branch from a sealed opaque plastic bag to ensure that vulnerability curves started from a maximally hydrated state. Subsequent measurements were then conducted successively throughout the dehydration process, with approximately eight to ten measurements per individual used to construct each curve. Branches were progressively dried through the bench dehydration technique^76^. Between each dehydration state, branches were bagged for a minimum of 1 h to equilibrate leaf and xylem water potentials. Leaf water potential (used as a proxy for xylem water potential following equilibration) was measured with a pressure chamber (PMS 1505D and PMS 1000, PMS instruments).

We used the exponential sigmoidal function of ref. ^88^ to calculate $\varPsi$_50_ for each species at each site:

2 $${\rm{PAD}}=\frac{100}{1+\exp \left[\frac{S}{25}({\varPsi }_{{\rm{x}}}-{\varPsi }_{50})\right]}$$

where *S* is the slope of the curve, *Ψ*_x_ is xylem water potential (MPa) and *Ψ*_50_ is *Ψ*_x_ corresponding to a PAD of 50%.

Following ref. ^89^, we computed *Ψ*_88_ as:

3 $${\varPsi }_{88}={\varPsi }_{50}-\frac{2}{\left(\frac{S}{25}\right)}$$

### ${\boldsymbol{\Psi }}$_dry_ and HSMs

To calculate how close Amazonian trees operate to critical embolism thresholds in nature, we measured in situ midday leaf water potentials during the peak of the dry season ($\varPsi$_dry_). Sampling campaigns closely corresponded with the time of most intense water deficit (Extended Data Fig. 2) and the year of sampling was not climatologically anomalous. We sampled three to six top-canopy fully expanded and sun-exposed leaves per individual (three individuals per species for 129 species in total across 11 sites) from 11:00 to 14:30. Parameter $\varPsi$_dry_ was measured with a pressure chamber (PMS 1505D and PMS 1000, PMS instruments) in situ immediately postsampling and the values of different leaves averaged per individual. In our protocol we tried to minimize the time spent between branch cutting and the leaf water potential measurement with the pressure chamber (around 3–5 min). We collected branches (40–60 cm in length, depending on the species and leaf size) that were fully exposed to light from the top part of the canopy (highest part that the climbers could reach), from apparently healthy and undamaged individuals. Telescopic shears (normally four to six poles, with total length of 5–7 m) were used to access and cut the branches. As soon as the branches hit the ground, the branches were bagged in a black and opaque plastic bag and transported to the pressure chamber, which was located inside the plot. We then collected three to six healthy and fully expanded leaves for each individual and immediately (after the cut) placed them into the pressure chamber. All of the processes were made as quickly as possible to avoid dehydration.

Because of pressure drops in transpiring leaves, we note that the water potentials measured are probably lower than the branch water potential values at the time of measurement. Apart from aseasonal ever-wet forests, which have no climatological dry season (monthly_precip_ < 100 mm), data collection took place in the peak of dry season (Extended Data Fig. 2) during what were climatically normal years. For each species at each site we calculated the HSM with respect to ${\varPsi }_{50}$ (HSM_50_), as the difference between species-level $\varPsi$_dry_, taken as the minimum $\varPsi$_dry_ value of all individuals for that species and $\varPsi$_50_. All $\varPsi$_dry_ measurements were made in climatologically normal years. (See Supplementary Table 8 for further information of sampling dates for each site). We also calculated the HSM with respect to ${\varPsi }_{88}$ (HSM_88_) for all sampled species.

### Wood density and leaf mass per area

We combined published and new field measurements of LMA and wood density to understand the power of these traits relative to HTs in predicting forest carbon balance. Stem wood density data (WD_stem_) were obtained from the Global Wood Density database^65,66^ and calculated as species mean values. We measured wood density at branch level (WD_branch_) using a water displacement method^90^. In this method, branch segments of about 25 mm length and 12 mm diameter were first cut and debarked. Samples were then placed in a recipient with filtered water to rehydrate for 24 h and subsequently weighed with a three-decimal scale. After this, the sample was oven-dried for 48–72 h at 70 °C and the dry weight measured with a balance. Wood density was then expressed as the ratio of wood dry mass and wood fresh volume (g cm^−3^). Branch wood density measurements were made in all sites except NVX. For this site, we used stem wood density values^65,66^ for each of our target species.

We measured LMA for all sampled species in each of the 11 sites. For this, all leaves were detached from a selected branch and a subsample of 10–20 leaves per branch were taken, numbered and scanned. All the other leaves were kept separate to be oven-dried. This was usually done as soon as possible after returning to the field station. When it was not possible to scan the leaves straight away, we placed all the detached leaves into a sealed plastic bag in the dark and stored them for no more than 24 h. After scanning, all leaves were oven-dried for 48–72 h at around 70 °C. Once dry, the subsampled numbered leaves were individually weighed and the non-numbered leaves were weighed together with a precision scale (three decimals). On the basis of the relationship between the fresh area and dry weight of individual leaves (from the subsampled 10–20 leaves) and having the dry weight of all the leaves of the branch, we estimated the fresh leaf area corresponding to the entire branch. The LMA was then calculated as the ratio of leaf dry mass to fresh area, expressed in g m^−2^. We then calculated basal area weighted mean values for all these traits for each site (Supplementary Table 2). The number of species sampled for each trait is shown in the Supplementary Table 9. Further leaf habit information of sampled species is provided in Supplementary Table 10.

### Water deficit affiliation

To describe Amazonian species-level biogeographical distributions, we used published WDA data^45^, which describes the spatial association of Amazonian tree species with climatological water availability. WDA was calculated as the mean climatological water deficit across inventory plots in which a species occurs weighted by its relative abundance in each of 513 forest plots broadly distributed in the western Neotropics^45^. More negative WDA values represent dry-affiliated species, whereas wet-affiliated species are represented by less negative WDA values.

### Forest dynamics data

We used long-term forest plots from the RAINFOR network^39^ to help understand the relationship between hydraulic attributes and stand-scale carbon dynamics. Thus, we computed AGB net change (*Δ*AGB, Mg ha^−1^ yr^−1^), annual aboveground wood production (AGWP, Mg ha^−1^ yr^−1^), annual AGB mortality (AGB_MORT_, Mg ha^−1^ yr^−1^), annual instantaneous stem mortality rate (% y^−1^) and woody biomass residence time (*τ*_w_) for the same forest plots sampled directly for HTs. For the two plots (MAN and TAP), for which we did not have access to forest dynamics data, we used information from a permanent RAINFOR network forest plot in the same landscape, with the most similar structure and species composition (BNT-01 and TAP-02, respectively; Supplementary Table 6) to our sampling plots. For CAX, we used published data by ref. ^91^ for the control plot. The other six plots are part of the RAINFOR network^39^, having been established by and/or monitored by RAINFOR partners (Supplementary Table 5). Plot data for these analyses were curated and obtained via the ForestPlots.net database^92,93^, for which standard quality control procedures are applied. We only included plots in the analysis that lacked a history of recent anthropogenic disturbance. For all forest dynamics analyses we excluded KEN plots because of a fire event that occurred in the region in 2004^68^ and may still be affecting biomass stocks and dynamics. Following previous studies^15,94^, plots smaller than 0.5 ha that were up to 1 km apart from each other were combined and treated as a single plot (for example, TAP-54, TAP-55, TAP-56 and TAP-57 treated as TAP-02, the plot we used to represent TAP). For each plot, we only included pre-2015 El Nino censuses and selected the census start date to be as consistent as possible across plots. For this we excluded pre-2000 measurements, apart from TAP plot for which censuses were available only from 1983 to 1995. For other plots, we used the earliest census available for this plot if data collection started after 2000 (VCR-02 plot, for example, which starts in 2003). We tried to ensure that biomass dynamics metrics used in the analyses represented at least 10 yr of total monitoring time per plot. If application of the 2000 start date for a given plot resulted in fewer than 10 yr of monitoring, we also included the census date immediately before 2000 (99 for BNT-02 plot, which we used to represent MAN) to ensure at least 10 yr of monitoring (Supplementary Table 5). The monitoring time used for the plots included in the analysis was on average 12.3 (s.d. = 2.5) yr. In RAINFOR plots, all live individuals of more than 10 cm in diameter at breast height (DBH) are repeatedly measured over time, using standardized protocols, with species identified and careful records kept of trees that die or recruit from one census to the next. AGB for each census per plot was computed using the ref. ^63^ equation for moist forests on the basis of tree diameter, wood density and height. As local height data were often unavailable, a Weibull equation with regionally varying coefficients was used to estimate height following ref. ^11^. Species-level wood density values from the Global Wood Density database^65,66^ were used to compute AGB, AGWP and AGB_MORT_. For each census, biomass values were calculated for all dicotyledonous trees in the plots above the 10 cm DBH cut-off and summed to give total stand-level biomass stocks.

We estimated annual *Δ*AGB (Mg ha^−1^ yr^−1^) for a given plot as the difference in AGB between the final and initial census used (AGB_final census_ − AGB_initial census_) divided by the monitoring length (Date_final census_ − Date_initial census_) in years. For each census interval per plot, we also computed annual AGWP (Mg ha^−1^ yr^−1^), following ref. ^95^, which encompasses (1) the sum of the growth of surviving trees, (2) the sum of AGB of new recruits, (3) the estimated sum of growth of unobserved recruits that dies and (4) the estimated sum of unobserved growth of initial trees that died, within a plot in a given census interval, divided by the census interval length (yr) (see also ref. ^94^). For each plot, we computed annual AGB_MORT_, including unobserved components, which is defined as the sum of the AGB of all dead trees, plus the estimated growth of recruits that died before they could be recorded in the second census and the sum of estimated unobserved growth of trees that died within an interval, divided by the census interval length^94^.

As AGB varies across sites it is useful to account for this when comparing sites. We therefore also computed relative *Δ*AGB (*Δ*AGB/AGB), relative AGWP and relative AGB_MORT_ by dividing absolute values by the time-weighted mean standing woody biomass across censuses per plot. Both absolute and relative values are presented in the Extended Data Figs. 7, 8 and 10 and Supplementary Table 4). We computed the annual instantaneous stem mortality rate (% y^−1^) following ref. ^67^:

4 $$\left(\frac{\left({\rm{ln}}(A)-{\rm{ln}}\left(B\right)\right)}{{\rm{census}}\,{\rm{interval}}}\right)\times 100$$

in which *A* is the number of stems per ha in the beginning of the census interval and *B* is the number of stems per ha that survived throughout the census interval. Owing to the sensitivity of these rates to census interval effects, we standardized them to a common census interval, following ref. ^96^. For all calculations above (AGB, AGWP, AGB_MORT_ and stem mortality) we used the BiomasaFP R package^97^. We calculated the time-weighted mean values of all these absolute and relative parameters (AGB, AGWP, AGB_MORT_ and stem mortality) to have one value per plot. We then calculated woody biomass residence time (*τ*_w_) as the ratio of the time-weighted mean standing woody biomass and the time-weighted mean annual biomass mortality^52^.

To test whether relationships between HSM_50_ and forest dynamics at plot level apply over landscape scales and to account for the influence of within- and among-plot stochasticity in dynamics, we also we used mean forest values of forest dynamics metrics across groups of plots (clusters) in the same landscape with similar structure and composition to plots sampled for hydraulic measurements (Supplementary Tables 5 and 6). For this cluster-level analysis, we excluded white-sand forests and permanently water-logged swamp forests because they are extreme edaphic habitats, known to have a more limited and edaphically specialized tree flora^98^. We also excluded forests lying within active floodplains of rivers because their flora is also distinctive and, like swamp forests, they have access to more water beyond that which is climatically determined. In total, we used data from 34 long-term monitoring plots (31.37 ha of forest). For this analysis, we used cluster mean forest dynamic values (instead of plot cluster weighted mean, for example) because plot area and monitoring length did not vary considerably within clusters (Supplementary Table 5). To account for sampling effort variation across cluster of forest plot, we tested if the residuals of the relationship between relative *Δ*AGB and HSM_50_ were related to cluster mean monitoring time (mean ± s.d. was 12.1 ± 1.8 yr) and cluster total area (3.9 ± 3.0 ha). No weights were assigned to each data point in the regression because we found no evidence of relationships between the residuals and sampling effort across clusters.

### Statistical analysis

To examine the distribution of HTs ($\varPsi$_50_, $\varPsi$_dry_ and HSM_50_) across Amazonian tree taxa (*N* = 129 species), trait values were averaged for species occurring at several sites. We conducted statistical analyses to investigate differences in species-level hydraulic trait values among different forest types and geographical regions and also to evaluate controls of water availability on basal area weighted mean HT across the study sites. To examine differences in HTs among forest types, we first grouped our 11 forest sites into three forest types, based on DSL: (1) ecotonal long DSL forests—DSL equal to 6 months, MAP and MCWD less than 1,600 and −470 mm, respectively; (2) intermediate DSL forests—DSL ranging from 5 to 2 months, MAP between 1,990 and 2,650 mm and MCWD varying from −288 to −184 mm; and (3) ever-wet aseasonal forests—DSL about 0 months, MAP and MCWD greater than 2,950 and −15 mm, respectively (Extended Data Fig. 1 and Supplementary Table 1). To test for statistical differences in HTs across forest types, we performed a one-way Kruskal–Wallis followed by a post hoc Mann–Whitney–Wilcoxon rank sum test. Western and central eastern Amazon forests have fundamentally different dynamics in that western Amazon forests are characterized by high growth and turnover whereas central eastern forests are associated with slow growth and turnover^35,36^. To test for differences between species in intermediate DSL sites in western Amazon (FEC and TAM) and central eastern Amazon (CAX, MAN and TAP), we performed Wilcoxon rank sum tests. Linear models were constructed to evaluate relationships between basal area weighted mean HT and MCWD (Supplementary Table 7). For all analyses, we use a significance level of 0.05.

To investigate if species biogeographical distributions are related to mean HT, we used SMA regressions with WDA as the response variable. Following Esquivel-Muelbert et al.^45^, we restricted our analysis to the western Amazon as these published WDA data are based entirely on species distributions within western Amazon, helping to control for the potentially confounding effects of differences in soil and forest dynamics across Amazonian regions. Our subsample for this analysis encompassed a total of 87 species distributed across aseasonal, intermediate DSL and ecotonal long DSL forests, with MAP across plots ranging from 1,390 to 3,170 mm. SMA regressions were performed using the smatr package^99^ in R.

Using our entire dataset across the Amazon, we evaluated whether HTs were better predictors of Amazon forest carbon balance than climatic factors or other leaf and wood traits. More specifically, we performed bivariate SMA models to investigate relationships between HTs ($\varPsi$_50_, $\varPsi$_dry_ and HSM_50_), climatic data (MCWD, MAP, DSL and MAT) and other functional traits (LMA, WD_stem_ and WD_branch_) versus long-term *Δ*AGB at plot level. We computed basal area weighted mean LMA, WD_branch_ and WD_stem_ data^65,66^. To account for the influence of multiple testing, we applied a Bonferroni correction to *P* values for bivariate regressions. SMA models were further conducted to examine the relationship between HSM_50_ versus absolute and relative values of AGB annual woody production, AGB annual mortality, stem mortality and residence time of woody biomass. Supplementary Table 5 presents summary information per plot and clusters. All presented analyses were performed in RStudio v.1.1.423 (ref. ^100^).

### Reporting summary

Further information on research design is available in the Nature Portfolio Reporting Summary linked to this article.

## Online content

Any methods, additional references, Nature Portfolio reporting summaries, source data, extended data, supplementary information, acknowledgements, peer review information; details of author contributions and competing interests; and statements of data and code availability are available at 10.1038/s41586-023-05971-3.

## Supplementary information

Supplementary Tables Supplementary Tables 1–10.

Reporting Summary

## Data Availability

The pan-Amazonian HT dataset ($\varPsi$_50_, $\varPsi$_dry_ and HSM_50_) and branch wood density per species per site, as well as forest dynamic and climate data per plot presented in this study are available as a ForestPlots.net data package at https://forestplots.net/data-packages/Tavares-et-al-2023. Basal area weighted mean LMA is shown in Supplementary Table 2. Species stem wood density data were obtained from Global Wood Density database^65,66^. Species WDA data were extracted from ref. ^45^.

## Code availability

The codes to recreate the main analyses and the main figures presented in this study are available as a ForestPlots.net data package at https://forestplots.net/data-packages/Tavares-et-al-2023.
