# Supplementary material for: Basin-wide variation in tree hydraulic safety margins predicts the carbon balance of Amazon forests
Source: Nature. 2023 Apr 26;617(7959):111–7. doi: 10.1038/s41586-023-05971-3 (PMC10156596; doi:10.1038/s41586-023-05971-3)
Supplement: Supplementary file 1 — Supplementary Tables 1–10. [file 41586_2023_5971_MOESM1_ESM.pdf]

nature portfolio

https://doi.org/10.1038/s41586-023-05971-3

Supplementary information

Basin-wide variation in tree hydraulic safety margins predicts the carbon balance of Amazon forests

In the format provided by the authors and unedited

Supplementary Information

Basin-wide variation in tree hydraulic safety margins predicts the carbon balance of Amazon forests

Julia Valentim Tavares[1,2*], Rafael S. Oliveira[3], Maurizio Mencuccini[4,5], Caroline Signori-Müller[6,7,2], Luciano Pereira[8,3], Francisco Carvalho Diniz[1], Martin Gilpin[1], Manuel J. Marca Zevallos[9], Carlos A. Salas Yupayccana[9], Martin Acosta[10], Flor M. Pérez Mullisaca[9], Fernanda de Vasconcellos Barros[6,11], Paulo Bittencourt[6,3], Halina Jancoski[12], Marina Corrêa Scalon[12,13], Beatriz Schwantes Marimon[12], Imma Oliveras Menor[14,15], Ben Hur Marimon Junior[12], Max Fancourt[1], Alexander Chambers-Ostler[1], Adriane Esquivel-Muelbert[16,17], Lucy Rowland[6], Patrick Meir[18,19], Antonio Carlos Lola da Costa[20], Alex Nina[21], Jesus M. B. Sanchez[9], Jose S. Tintaya[9], Rudi S. C. Chino[21], Jean Baca[22], Leticia Fernandes[9], Edwin R. M. Cumapa[20], João Antônio R. Santos[9], Renata Teixeira[9], Ligia Tello[22], Maira T. M. Ugarteche[23,24], Gina A. Cuellar[23,24], Franklin Martinez[23,24], Alejandro Araujo-Murakami[23,24], Everton Almeida[25], Wesley Jonatar Alves da Cruz[12], Jhon del Aguila Pasquel[26], Luís Aragão[27], Timothy R. Baker[1], Plinio Barbosa de Camargo[28], Roel Brienen[1], Wendeson Castro[29,30], Sabina Cerruto Ribeiro[31], Fernanda Coelho de Souza[32], Eric G. Cosio[33], Nallaret Davila Cardozo[26], Richarlly da Costa Silva[10,34], Mathias Disney[35], Javier Silva Espejo[9,36], Ted Feldpausch[6], Leandro Ferreira[37], Leandro Giacomin[38,39,40], Niro Higuchi[41], Marina Hirota[42,3], Euridice Honorio[26], Walter Huaraca Huasco[14], Simon Lewis[1,36], Gerardo Flores Llampazo[43,26], Yadvinder Malhi[14], Abel Monteagudo Mendoza[9,44], Paulo Morandi[12], Victor C. Moscoso[9,44], Robert Muscarella[2], Deliane Penha[40], Mayda Cecília Rocha[45], Gleicy Rodrigues[42], Ademir R. Ruschel[46], Norma Salinas[14,33], Monique Schlickmann[46], Marcos Silveira[47], Joey Talbot[48], Rodolfo Vásquez[44], Laura Vedovato[6], Simone Aparecida Vieira[49], Oliver L. Phillips[1], Emanuel Gloor[1], David R. Galbraith[1]

Table 1. Sampling design summary: Site environmental characteristics

Forest type	Biogeographic region	Site	MCWD (mm)	MAP (mm)	DSL (months)	MAT (°C)
Aseasonal	Western Amazon	ALP1	-23.11	2956.01	0	26.13
		ALP2	-23.28	2956.01	0	26.13
		SUC	-15.09	3178.95	0	26.33
Inter DSL	Western Amazon	FEC	-288.74	1995.77	5	25.29
		TAM	-195.26	2653.33	2	25.22
	C.Eastern Amazon	MAN	-184.26	2464.54	2	26.56
		CAX	-256.09	2547.44	4	26.23
		TAP	-250.14	2047.78	5	25.98
Long DSL	Southern Amazon	NVX	-643.30	1539.26	6	24.81
	Western Amazon	KEN1	-472.45	1395.35	6	24.53
		KEN2	-476.00	1395.35	6	24.53

Maximum climatological water deficit (MCWD); Mean annual precipitation (MAP); Dry season length (DSL) = number of months with <100mm precipitation; Mean annual temperature (MAT).

Table 2. Sampling design summary: Site biotic characteristics.

Forest type	Biog. region	Site	% Basal area sampled for p50	p50	p88	% Basal area sampled for Psi	Psi_min	HSM50	HSM88	% Basal area sampled for LMA	LMA	WD branch
Aseasonal	Western Amazon	ALP1	21.88	-1.97	-3.28	21.88	-0.96	1.02	2.33	26.21	109.01	0.58
		ALP2	27.04	-1.44	-2.26	26.66	-0.71	0.72	1.56	27.32	131.54	0.57
		SUC	20.78	-1.29	-2.09	20.78	-0.97	0.32	1.13	23.33	116.44	0.57
Inter DSL	Western Amazon	FEC	56.26	-2.26	-3.85	41.02	-2.62	-0.32	1.33	58.85	113.69	0.65
		TAM	59.36	-1.54	-2.57	50.55	-1.52	-0.01	1.04	59.36	120.72	0.66
	C.Eastern Amazon	MAN	13.69	-2.07	-3.30	13.69	-1.48	0.59	1.81	11.66	240.59	0.67
		CAX	25.50	-2.18	-4.17	25.50	-2.51	-0.32	1.66	19.98	101.51	0.62
		TAP	37.03	-2.75	-4.63	26.28	-1.82	1.04	2.76	41.47	NA	0.69
Long DSL	Southern Amazon	NVX	51.38	-2.95	-4.10	51.38	-3.95	-1.00	0.15	51.38	104.17	0.72
	Western Amazon	KEN1	68.78	-2.44	-3.86	68.78	-2.10	0.34	1.76	NA	NA	0.57
		KEN2	57.50	-2.27	-4.00	52.93	-3.49	-1.12	0.54	NA	NA	0.70

For all variables we use basal area weighted mean values per plot.

Table 3 Results of the standard major axis regressions (SMA) of the bivariate relationship between *relative aboveground net biomass changes and basal-area-weighted-mean vegetation traits* and climatic variables across Amazonian forest plots. Lower and upper CI represent the lower and upper 95% confidence intervals for the slope and intercept. Significant adjusted *p* values after Bonferroni correction are shown in bold.

	Model	N	r2	pval	Slope	Slope lowCI	Slope highCI	Inter	Inter lowCI	Inter highCI	p value Bonferroni
Rel ΔAGB~	**HSM_{50}**	9	**0.70**	0.01	0.90	0.56	1.45	-0.04	-0.35	0.28	**0.05**
	Ψ_{dry}	9	0.65	0.01	0.60	0.36	1.00	1.28	0.61	1.95	0.08
	MCWD	9	0.53	0.03	0.00	0.00	0.01	0.83	0.27	1.39	0.23
	MAP	9	0.39	0.07	0.00	0.00	0.00	-2.73	-4.80	-0.65	0.66
	MAT	9	0.34	0.10	1.04	0.53	2.05	-26.84	-46.38	-7.29	0.87
	Ψ_{50}	9	0.22	0.20	1.10	0.53	2.26	2.42	0.56	4.28	1.00
	Branch WD	9	0.17	0.27	-11.38	-23.94	-5.41	7.43	1.49	13.37	1.00
	Stem WD	9	0.06	0.52	-9.65	-21.13	-4.41	6.45	0.97	11.92	1.00
	LMA	9	0.01	0.85	0.01	0.01	0.03	-1.62	-3.37	0.13	1.00

Table 4 Results of the standard major axis models (SMA) between basal-area-weighted mean HSM_{50} in relation to plot and cluster-level metrics of forest dynamics (Extended Data Figures 7, 8, 10). Significant *p* values are shown in bold. Lower and upper CI represent the lower and upper 95% confidence intervals for the slope and intercept. Further cluster information and the observation period used for each cluster is available on SM Table 5.

Analysis level	X	Y	n	r2	pval	Slope	Slope lowCI	Slope highCI	Inter	Inter lowCI	Inter highCI	Reference figure
Plot	HSM_{50}	**REL ΔAGB**	9	**0.7**	**0.01**	0.9	0.56	1.45	-0.04	-0.35	0.28	ED Fig 7
		REL AGB_{MORT}	9	0.26	0.16	-1.37	-2.78	-0.68	2.13	1.31	2.95	
		REL AGWP	9	0	0.94	0.95	0.42	2.12	1.78	0.99	2.57	
		Woody Biomass Residence time	9	0.18	0.25	69.66	33.25	145.95	57.98	12.69	103.27	
		**ΔAGB**	9	**0.67**	**0.01**	2.37	1.45	3.89	0.17	-0.71	1.05	
		AGB_{MORT}	9	0.16	0.29	-2.53	-5.35	-1.2	5.37	3.69	7.06	
		**Stem Mortality**	9	**0.47**	**0.04**	-1.64	-3.03	-0.89	2.55	1.75	3.35	
		AGWP	9	0.25	0.17	1.82	0.89	3.7	5.11	4	6.21	
Cluster	HSM_{50}	**REL ΔAGB**	8	**0.68**	**0.01**	1.05	0.61	1.8	-0.07	-0.47	0.34	ED Fig 8

		REL AGB_{MORT}	8	0.27	0.18	-1.45	-3.13	-0.67	2.01	1.08	2.93	
		REL AGWP	8	0.02	0.76	0.75	0.31	1.8	1.78	1.13	2.42	
		Woody Biomass Residence time	8	0.31	0.15	64.08	30.08	136.48	71	31.46	110.54	
		**ΔAGB**	8	**0.67**	**0.01**	2.96	1.71	5.12	0.07	-1.11	1.24	
		AGB_{MORT}	8	0.3	0.16	-3.1	-6.63	-1.44	5.4	3.46	7.33	
		Stem Mortality	8	0.47	0.06	-1.43	-2.81	-0.73	2.23	1.48	2.97	
		AGWP	8	0.28	0.18	1.37	0.63	2.95	5.25	4.38	6.12	
		**REL ΔAGB**	9	**0.51**	**0.03**	0.82	0.45	1.49	-1.09	-1.97	-0.2	
		REL AGB_{MORT}	9	0.36	0.09	-1.25	-2.43	-0.64	3.73	2.19	5.28	
Plot	HSM_{88}	REL AGWP	9	0.05	0.58	-0.86	-1.9	-0.39	3.31	1.97	4.66	ED Fig 10
		**Woody Biomass Residence time**	9	**0.48**	**0.04**	63.4	34.51	116.5	-23.17	-93.49	47.15	
		**ΔAGB**	9	**0.56**	**0.02**	2.16	1.23	3.79	-2.59	-4.78	-0.4	

		AGB_{MORT}	9	0.18	0.25	-2.3	-4.82	-1.1	8.32	5.06	11.57	
		**Stem Mortality**	9	**0.68**	**0.01**	-1.49	-2.43	-0.92	4.46	3.17	5.74	
		AGWP	9	0.13	0.34	1.65	0.77	3.53	2.99	0.57	5.41	
Cluster	HSM_{88}	REL ΔAGB	8	0.46	0.07	0.94	0.47	1.86	-1.30	-2.46	-0.14	No figure
		REL AGB_{MORT}	8	0.29	0.17	-1.30	-2.79	-0.60	3.71	1.87	5.56	
		REL AGWP	8	0.01	0.81	-0.67	-1.61	-0.28	2.88	1.70	4.05	
		**Woody Biomass Residence time**	8	**0.56**	**0.03**	57.45	30.92	106.73	-4.49	-67.57	58.59	
		**ΔAGB**	8	**0.49**	**0.05**	2.65	1.36	5.16	-3.42	-6.59	-0.25	
		AGB_{MORT}	8	0.21	0.25	-2.78	-6.17	-1.25	9.04	4.87	13.22	
		**Stem Mortality**	8	**0.63**	**0.02**	-1.28	-2.28	-0.72	3.91	2.63	5.20	
		AGWP	8	0.21	0.25	1.22	0.55	2.72	3.64	1.79	5.49	

Annual ΔAGB: annual net biomass change, calculated as the difference in aboveground biomass between the final and initial census used (AGBfinal census – AGBinitial census) divided by the monitoring length (Datefinal census – Dateinitial census) in years.; AGWP: Annual aboveground wood productivity, defined as the sum of the biomass growth of surviving trees >10 cm DBH, new recruits > 10 cm DBH and the unobserved components (growth of unobserved recruits and growth of trees that died), within a plot in a given census interval[91], divided by the census interval length; AGB_{MORT}: Annual aboveground biomass mortality, which is the sum of the AGB of all dead trees and the unobserved components (growth of recruits that died and growth of trees that died within an interval), divided by the census interval length[91]; Stem Mort: Annual instantaneous stem mortality rate (See methods Equation 4)[64]; tau: Residence time of

woody biomass calculated as the ratio of mean standing biomass to mean biomass mortality rate[43]. REL Annual ΔAGB: ΔAGB/AGB ; REL Annual AGWP: Relative annual AGB wood productivity, defined as AGWP divided by the time-weighted mean standing woody biomass (AGB) across censuses per plot; REL Annual AGB_{MORT}: Relative annual biomass mortality (AGB_{MORT}/AGB). All these parameters were calculated for each census interval and we calculated time-weighted mean to have one value per plot. KEN plots were excluded from all forest dynamics analyses because of a fire event that occurred in the region in 2004[63] and may still be affecting biomass accrual.

Table 5. Summary of forest plots and clusters of forest plots used to investigate changes in net biomass.

Cluster	Mean monitoring time (Yr)	Total cluster area (ha)	Plot Code	Date initial census	Date final census	Monitoring time (Yr)	Plot area (ha)
ALP			ALP-02	2001.03	2015.12	14.09	0.44
ALP	14.09	1.32	**ALP-11**	2001.03	2015.12	14.09	0.48
ALP			**ALP-12**	2001.03	2015.12	14.09	0.4
CAX			CAX-01	1999.50	2009.95	10.45	1
CAX			CAX-02	1999.50	2009.95	10.45	1
CAX			CAX-06	2004.61	2009.95	5.35	1
CAX			TEC-01	2002.87	2012.81	9.94	1
CAX			TEC-02	2003.19	2012.81	9.63	1
CAX	9.07	9.81	TEC-03	2003.22	2012.82	9.60	1
CAX			TEC-04	2003.31	2012.82	9.51	1
CAX			TEC-05	2003.48	2012.81	9.34	1
CAX			TEC-06	2003.33	2012.81	9.48	1
CAX			**CAX-control**	2001.00	2008.00	7.00	0.81
FEC	10.90	2	**FEC-01**	2000.86	2013.50	12.64	1
FEC			RFH-01	2004.33	2013.49	9.15	1
MAN			**BNT-01***	1999.54	2010.53	10.99	1
MAN	10.99	3	BNT-02	1999.54	2010.53	10.99	1
MAN			BNT-04	1999.54	2010.53	10.99	1
SUC			**SUC-01**	2001.06	2015.51	14.45	1
SUC	14.42	4	**SUC-02**	2001.07	2015.53	14.46	1
SUC			SUC-04	2001.16	2015.53	14.37	1
SUC			SUC-05	2001.12	2015.52	14.40	1
TAM			TAM-01	2000.60	2014.70	14.10	1
TAM			TAM-02	2000.58	2014.70	14.13	1
TAM			**TAM-05**	2000.56	2014.71	14.15	1
TAM	12.09	7	TAM-06	2000.55	2014.67	14.12	1
TAM			TAM-07	2003.72	2014.70	10.98	1
TAM			TAM-08	2001.53	2014.69	13.16	1
TAM			TAM-09	2010.69	2014.67	3.99	1
TAP			TAP-01	1983.50	1995.50	12.00	1
TAP	12.00	3	**TAP-02***	1983.50	1995.50	12.00	1
TAP			TAP-03	1983.50	1995.50	12.00	1
VCR	12.96	1.24	VCR-01	2001.50	2015.50	14.01	0.64
VCR			**VCR-02**	2003.59	2015.50	11.92	0.6

Plots where hydraulic traits were sampled are shown in bold. Plots in bold marked with asterisks (BNT-01 and TAP-02) are the plots in the same landscape, with the similar structure and the most similar species composition to TAP and MAN plots where hydraulic traits were measured and for which we did not have access to forest dynamics data. We used BNT-01 and TAP-02 to represent forest dynamics of TAP and MAN (See SI Table 6).

Table 6. Species composition similarity between plots on which we sampled hydraulic traits, leaf mass per area and wood density and other plots in the same cluster, in terms of abundance and basal area.

Cluster	Plot Code	Sum of the relative abundance of the species which hydraulic traits were measured (in terms of number of stem) (%)	Sum of the relative dominance of the species which hydraulic traits were measured (in terms of basal area) (%)	Hydraulic Traits Sampled?
**ALP**	**ALP-11**	**26.4**	**23.49**	This study
**ALP**	**ALP-02**	**19.01**	**16.61**	This study
MAN	BNT-01	13.27	15.43	
MAN	BNT-02	11.99	7.54	
MAN	BNT-04	11.5	7.1	
**MAN**	**MAN - Barros et al. 2019**		**13.69**	Barros et. al 2019
**CAX control**	**CAX - Bittencourt et al 2020**		**25.50**	Bittencourt et al 2020
CAX	TEC-01	27.32	21.07	
CAX	TEC-02	20.62	16.25	
CAX	TEC-03	17.51	10.39	
CAX	TEC-04	25.79	17.18	
CAX	TEC-05	21.38	17.3	
CAX	TEC-06	33.87	26.99	
CAX	CAX-01	28.88	21.67	
CAX	CAX-02	27.27	25.44	
CAX	CAX-06	23.28	7.97	
**FEC**	**FEC-01**	**45.83**	**27.64**	This study
FEC	RFH-01	20.61	13.18	
**KEN**	**KEN-01**	**71.29**	**45.11**	This study
**KEN**	**KEN-02**	**62.22**	**54.09**	This study
**SUC**	**SUC-01**	**38.92**	**24.92**	This study
**SUC**	**SUC-02**	**30.41**	**21.89**	This study
SUC	SUC-04	32.31	24.3	
SUC	SUC-05	32.22	21.45	
TAM	TAM-01	22.01	23.87	
TAM	TAM-02	35.5	23.3	
**TAM**	**TAM-05**	**56.61**	**45.3**	This study

TAM	TAM-06	14.19	10.42	
TAM	TAM-07	57.76	37.39	
TAM	TAM-08	28.46	28.84	
TAM	TAM-09	30.78	24.44	
**TAP**	**TAP - Brum et al 2018**		**37.03**	Brum et al 2018..
TAP	TAP-01	20.04	10.23	
TAP	TAP-02	21.73	11.9	
TAP	TAP-03	20.95	11.82	
NVX	VCR-01	93.5	81.92	
**NVX**	**VCR-02**	**52.19**	**60.42**	Jancoski et al. (unplublished)

Table 7. Sensitivity of relationships between basal area weighted mean hydraulic traits and climate data from alternative sources: mean annual precipitation (MAP) and maximum cumulative water deficit (MCWD). The relationships were tested based on precipitation datasets from TRMM and from the Climate Research Unit (CRU). MCWD was further estimated using site-specific values from MODIS 16 and ERA-15 and with the default method in the literature of assuming an evapotranspiration demand of 100 mm $month^{-1}$
The table is sorted by AIC for each hydraulic trait evaluated (Ψ_{50}, HSM_{50} and Ψ_{dry}).

Hydraulic trait	Models	Residual standard error	Multiple R-squared	Adjusted R-squared	F-statistic	p-value	Intercept	Intercept SE	Slope	Slope SE	AIC
Ψ_{50}	~MCWD_TRMM_MOD16	0.26	0.78	0.75	31.45	**0.000**	-1.44	0.14	0.00	0.00	5.46
	~MCWD_TRMM_100	0.30	0.70	0.67	21.17	**0.001**	-1.61	0.14	0.00	0.00	8.69
	~MCWD_CRU_100	0.32	0.67	0.63	18.13	**0.002**	-1.58	0.16	0.00	0.00	9.86
	~MCWD_CRU_MOD16	0.32	0.66	0.63	17.80	**0.002**	-1.47	0.18	0.00	0.00	9.99
	~MAP_TRMM	0.34	0.63	0.59	15.55	**0.003**	-3.58	0.39	0.00	0.00	10.96
	**~MCWD_TRMM_ERA5**	**0.34**	**0.63**	**0.59**	**15.28**	**0.004**	**-1.59**	**0.17**	**0.00**	**0.00**	**11.08**
	~MAP_CRU	0.35	0.60	0.55	13.41	**0.005**	-3.32	0.35	0.00	0.00	11.96
	~MCWD_CRU_ERA5	0.38	0.54	0.49	10.57	**0.010**	-1.60	0.19	0.00	0.00	13.45
HSM_{50}	**~MCWD_TRMM_ERA5**	**0.54**	**0.52**	**0.47**	**9.89**	**0.012**	**0.78**	**0.27**	**0.00**	**0.00**	**21.53**
	~MCWD_TRMM_100	0.57	0.47	0.41	8.06	**0.019**	0.68	0.27	0.00	0.00	22.65

	~MCWD_CRU_ERA5	0.60	0.42	0.35	6.45	**0.032**	0.74	0.31	0.00	0.00	23.74
	~MCWD_CRU_100	0.62	0.38	0.31	5.42	**0.045**	0.67	0.31	0.00	0.00	24.50
	~MCWD_TRMM_MOD16	0.63	0.35	0.28	4.88	0.054	0.74	0.35	0.00	0.00	24.91
	~MAP_TRMM	0.64	0.33	0.25	4.38	0.066	-1.39	0.74	0.00	0.00	25.32
	~MCWD_CRU_MOD16	0.67	0.27	0.18	3.26	0.105	0.68	0.38	0.00	0.00	26.28
	~MAP_CRU	0.67	0.26	0.18	3.21	0.107	-1.03	0.67	0.00	0.00	26.33
	**~MCWD_TRMM_ERA5**	**0.44**	**0.84**	**0.82**	**47.89**	**0.000**	**-0.81**	**0.22**	**0.00**	**0.00**	**17.06**
	~MCWD_TRMM_100	0.44	0.84	0.82	47.39	**0.000**	-0.93	0.21	0.01	0.00	17.16
	~MCWD_TRMM_MOD16	0.53	0.77	0.74	30.18	**0.000**	-0.68	0.29	0.00	0.00	21.16
	~MCWD_CRU_100	0.57	0.74	0.71	24.99	**0.001**	-0.91	0.28	0.01	0.00	22.73
Ψ_{dry}	~MCWD_CRU_ERA5	0.61	0.70	0.67	21.00	**0.001**	-0.86	0.31	0.00	0.00	24.10
	~MAP_TRMM	0.64	0.67	0.64	18.53	**0.002**	-5.07	0.73	0.00	0.00	25.05
	~MCWD_CRU_MOD16	0.68	0.63	0.59	15.16	**0.004**	-0.77	0.38	0.00	0.00	26.48
	~MAP_CRU	0.71	0.59	0.55	13.00	**0.006**	-4.45	0.71	0.00	0.00	27.51
	~MAT	1.06	0.09	-0.01	0.91	0.364	-10.15	8.50	0.31	0.32	36.28

Table 8. Hydraulic traits sampling periods per site

Forest type	Site	P50	Psi	LMA	WD branch
Aseasonal	ALP1	Out 2017	Out 2017	Out 2017	Out 2017
	ALP2	Out 2017	Out 2017	Out 2017	Out 2017
	SUC	Nov 2017	Nov 2017	Nov 2017	Nov 2017
Inter DSL	CAX	Set/Out 2016	Set/Out 2016	Set/Out 2016	Set/Out 2016
	FEC	April 2017	Jul/Aug 2017	April 2017	April 2017
	MAN	Aug 2015	Aug 2016	Aug 2015	Aug 2015
	TAM	Jan/Feb 2017	Set 2017	Jan/Feb 2017	Jan/Feb 2017
	TAP	Nov 2014	Dec 2014	Sep 2021	Nov 2014
Long DSL	NVX	March 2017	Aug 2016	Aug 2018	Not collected
	KEN1	March 2017	Aug/Sep 2017<br>July 2018	March 2017	March 2017
	KEN2	March 2017	Aug/Sep 2017<br>July 2018	March 2017	March 2017

Table 9. Number of species sampled for each trait per site

Forest type	Site	Ψ_{50}	Ψ_{dry}	HSM_{50}	LMA	WD branch
Aseasonal	ALP1	14	14	14	17	17
	ALP2	11	10	10	12	12
	SUC	23	23	23	24	24
Inter DSL	CAX	18	18	18	18	18
	FEC	19	15	15	21	20
	MAN	17	17	17	17	17
	TAM	26	21	21	26	26
	TAP	10	7	7	38	10
Long DSL	NVX	7	7	7	7	7
	KEN1	11	11	11	NA	13
	KEN2	12	11	11	NA	13

KEN plots were excluded from all forest dynamics analyses because of a fire event that occurred in the region in 2004[65] and may still be affecting biomass accrual.

Table 10. Leaf habit information of sampled species per site. Top rows indicate the total number of species from which hydraulic traits were sampled and the total sampled basal area. Leaf habit data were obtained from literature and based on field observations from Alessandro Araujo-Murakami.

	Number of species (HSM_{50})	Percentage of sampled basal area (HSM_{50})
**ALP1**	**17**	**27.16**
Deciduous	1	0.52
Evergreen	7	8.59
NA	8	17.39
Semideciduous	1	0.66
**ALP2**	**12**	**27.32**
Deciduous	1	0.95
Evergreen	5	16.40
NA	6	9.97
**CAX**	**18**	**25.50**
Evergreen	8	14.98
NA	9	10.49
Semideciduous	1	0.03
**FEC**	**21**	**59.43**
Deciduous	7	20.83
Evergreen	6	22.89
NA	6	13.12
Semideciduous	2	2.59
**KEN1**	**13**	**75.16**
Deciduous	2	4.54
Evergreen	2	27.58
Semideciduous	9	43.04
**KEN2**	**13**	**59.22**
Deciduous	8	50.60
Semideciduous	5	8.63
**MAN**	**17**	**13.69**
Evergreen	5	4.41
NA	11	8.08
Semideciduous	1	1.20
**NVX**	**7**	**51.38**
Deciduous	1	8.78
Evergreen	4	27.97
NA	2	14.63
**SUC**	**24**	**23.90**

Deciduous	2	4.80
Evergreen	6	4.72
NA	16	14.37
**TAM**	**26**	**59.36**
Deciduous	2	2.36
Evergreen	14	24.91
NA	7	26.57
Semideciduous	3	5.52
**TAP**	**10**	**37.03**
Evergreen	7	26.18
NA	3	10.85
