# Supplementary material for: Basin-wide variation in tree hydraulic safety margins predicts the carbon balance of Amazon forests
Source: Nature. 2023 Apr 26;617(7959):111–7. doi: 10.1038/s41586-023-05971-3 (PMC10156596; doi:10.1038/s41586-023-05971-3)
Supplement: Supplementary file 2 — Reporting Summary [file 41586_2023_5971_MOESM2_ESM.pdf]

nature portfolio

Corresponding author(s): Julia Valentim Tavares

Last updated by author(s): 2022/12/12

Reporting Summary

Nature Portfolio wishes to improve the reproducibility of the work that we publish. This form provides structure for consistency and transparency in reporting. For further information on Nature Portfolio policies, see our Editorial Policies and the Editorial Policy Checklist.

Statistics

For all statistical analyses, confirm that the following items are present in the figure legend, table legend, main text, or Methods section.

n/a	Confirmed	
☐	☒	The exact sample size (*n*) for each experimental group/condition, given as a discrete number and unit of measurement
☐	☒	A statement on whether measurements were taken from distinct samples or whether the same sample was measured repeatedly
☐	☒	The statistical test(s) used AND whether they are one- or two-sided<br>*Only common tests should be described solely by name; describe more complex techniques in the Methods section.*
☐	☒	A description of all covariates tested
☐	☒	A description of any assumptions or corrections, such as tests of normality and adjustment for multiple comparisons
☐	☒	A full description of the statistical parameters including central tendency (e.g. means) or other basic estimates (e.g. regression coefficient) AND variation (e.g. standard deviation) or associated estimates of uncertainty (e.g. confidence intervals)
☐	☒	For null hypothesis testing, the test statistic (e.g. *F*, *t*, *r*) with confidence intervals, effect sizes, degrees of freedom and *P* value noted<br>*Give P values as exact values whenever suitable.*
☒	☐	For Bayesian analysis, information on the choice of priors and Markov chain Monte Carlo settings
☒	☐	For hierarchical and complex designs, identification of the appropriate level for tests and full reporting of outcomes
☒	☐	Estimates of effect sizes (e.g. Cohen's *d*, Pearson's *r*), indicating how they were calculated

Our web collection on statistics for biologists contains articles on many of the points above.

Software and code

Policy information about availability of computer code

Data collection

To quantify xylem resistance to embolism of Amazonian trees species we constructed xylem vulnerability curves using the pneumatic method of Pereira et al. (2016). To generate these curves, we used the open source R software version 3.6.3, RStudio version 1.1.423 and R script from Bittencourt et al., 2018 available at: https://en.bio-protocol.org/CN/e3059#biaoti25711.

To calculate leaf we used the ImageJ software (Schneider, Rasband & Eliceiri, 2012).

References:
-Pereira, L., Bittencourt, P.R., Oliveira, R.S., Junior, M.B., Barros, F.V., Ribeiro, R.V. and Mazzafera, P. Plant pneumatics: stem air flow is related to embolism–new perspectives on methods in plant hydraulics. New Phytologist, 211(1), pp.357-370 (2016).
-Bittencourt, P., Pereira, L. & Oliveira, R. Pneumatic Method to Measure Plant Xylem Embolism. Bio-Protocol 8, 1–14 (2018).
-Schneider, C. A., Rasband, W. S., & Eliceiri, K. W. (2012). NIH Image to ImageJ: 25 years of image analysis. Nature Methods, 9(7), 671–675.

Data analysis

All data analysis was performed using the open source R software version 3.6.3 and RStudio version 1.1.423 and packages dplyr (1.0.6) and tidyr (1.1.3) to manage datasets. Standardized major axis (SMA) regressions were perfumed using the smatr package (3.4-8). We created and plotted the figures using ggplot2 (3.3.4) and egg (0.4.5). We used package modelr (0.1.8) and purrr (0.3.4) to create 95% bootstrapped confidence interval of best fit line from the Standard major axis model. We used the biomasaFP package to calculate forest dynamic parameters (i.e., AGB, AGWP, AGBMORT and stem mortality).

References:
-R Core Team (2020). R: A language and environment for statistical computing. R Foundation for Statistical Computing, Vienna, Austria. URL https://www.R-project.org/.
-RStudio Team. RStudio: Integrated Development for R. Boston, MA: RStudio, Inc. (2016). Available at: http://www.rstudio.com/.
-Hadley Wickham, Romain François, Lionel Henry and Kirill Müller (2021). dplyr: A Grammar of Data Manipulation. R package version 1.0.6.
-Hadley Wickham (2021). tidyr: Tidy Messy Data. R package version 1.1.3.

-Warton, David I., Duursma, Remko A., Falster, Daniel S. and Taskinen, Sara (2012) smatr 3 - an R package for estimation and inference about allometric lines. Methods in Ecology and Evolution, 3(2), 257-259
-H. Wickham. ggplot2: Elegant Graphics for Data Analysis. Springer-Verlag New York, 2016.
-Baptiste Auguie (2019). egg: Extensions for 'ggplot2': Custom Geom, Custom Themes, Plot Alignment, Labelled Panels, Symmetric Scales, and Fixed Panel Size. R package version 0.4.5.
-Wickham H (2022). modelr: Modelling Functions that Work with the Pipe. https://modelr.tidyverse.org, https://github.com/tidyverse/modelr.
-Henry L, Wickham H (2022). purrr: Functional Programming Tools. http://purrr.tidyverse.org
-Lopez-Gonzalez, G., Sullivan, M. J. P. & Baker, T. R. BiomasaFP: Tools for analysing data downloaded from ForestPlots. net. R package version. 1(1),

For manuscripts utilizing custom algorithms or software that are central to the research but not yet described in published literature, software must be made available to editors and reviewers. We strongly encourage code deposition in a community repository (e.g. GitHub). See the Nature Portfolio guidelines for submitting code & software for further information.

Data

Policy information about availability of data

All manuscripts must include a data availability statement. This statement should provide the following information, where applicable:

- Accession codes, unique identifiers, or web links for publicly available datasets
- A description of any restrictions on data availability
- For clinical datasets or third party data, please ensure that the statement adheres to our policy

The pan-Amazonian hydraulic traits data set (ψ50, ψdry, HSM50) and branch wood density per species per site, as well as forest dynamic and climate data per plot presented in this study will be available, before the publication, as a ForestPlots.net data package at https://forestplots.net/data-packages/Tavares-et-al-2023. Basal-area-weighted mean leaf mass per area is displayed in SM. Table 2. Species stem wood density data were obtained from Global Wood Density database (Chave et al., 2009; Zanne et al., 2009). Species water deficit affiliation data were extracted from Esquivel-Muelbert et al. (2017).

References:
-Chave, J. et al. Towards a worldwide wood economics spectrum. Ecol. Lett. 12, 351–366 (2009).
-Zanne, A. E. et al. Data from: Towards a worldwide wood economics spectrum. Ecology Letters (2009).
-Fyllas, N. M. et al. Basin-wide variations in foliar properties of Amazonian forest: phylogeny, soils and climate. Biogeosciences 6, 2677–2708 (2009).
-Esquivel-Muelbert, A. et al. Seasonal drought limits tree species across the Neotropics. Ecography (Cop.). 40, 618–629 (2017).
-Fick, S. E. & Hijmans, R. J. WorldClim 2: new 1-km spatial resolution climate surfaces for global land areas. Int. J. Climatol. 37, 4302–4315 (2017).
-Harris, I., Jones, P. D., Osborn, T. J. & Lister, D. H. Updated high-resolution grids of monthly climatic observations - the CRU TS3.10 Dataset. Int. J. Climatol. (2014).

Field-specific reporting

Please select the one below that is the best fit for your research. If you are not sure, read the appropriate sections before making your selection.

☐ Life sciences ☐ Behavioural & social sciences ☒ Ecological, evolutionary & environmental sciences

For a reference copy of the document with all sections, see nature.com/documents/nr-reporting-summary-flat.pdf

Ecological, evolutionary & environmental sciences study design

All studies must disclose on these points even when the disclosure is negative.

Study description

In this study, we present the first pan-Amazonian dataset of key tree hydraulic traits (embolism resistance: ψ50, hydraulic safety margins: HSM50 and minimum in situ leaf water potential: ψmin,) from 129 species across 11 forests plots, which span the entire Amazon precipitation gradient and vary among ever-wet aseasonal, climatically seasonal and ecotonal forests types and assess variation in drought sensitivity across Amazonia. This this new large-scale dataset combined with long-term forest inventory (RAINFOR network - ForestPlots.net et al., 2021) information allowed us to evaluate the ability of these traits to predict biogeographical distributions of Amazon species and long-term forest biomass accumulation under climate change. For each plot, we computed forest dynamics information (relative and absolute values of aboveground biomass net change, aboveground biomass mortality and aboveground wood production, as well as stem mortality and residence time of woody biomass).

Reference:
-ForestPlots.net et al. Taking the pulse of Earth ’ s tropical forests using networks of highly. 260, (2021).

Research sample

To caracterize spatial variation of hydraulic traits across amazonian forests, we combined new data collection and published data from Brum et al. (2018), Barros et al. (2019) and Bittencourt et al. (2020) which used the same methodology and sampling design of this present study. For our data collection, 8 forest plots were selected to represent tree communities across a wide precipitation gradient, ranging from ecotonal forests in the south of the basin to ever-wet forests in the northwest. We selected forest on the western and southern Amazon due to the lack of information for these regions, as most hydraulic traits information for the intere domain is given from central-eastern Amazon. Our data collection was nested within RAINFOR permanent inventory plot network (Forestplots.net et al 2021). For each site, we extracted information about species composition and location from ForestPlots.net database (ForestPlots.net et al., 2021) and focused our sampling on the most dominant adult canopy and sub-canopy tree species, in terms of basal area. Tree botanical identifications were carried out by RAINFOR partners and botanical vouchers are deposited in Amazon state herbaria (AMAZ, CUZ, HOXA, INPA, UFACPZ, USZ). In total, our hydraulic traits dataset consist in 129 tree species (including published data quoted above), 88 genera and 35 families distributed across 11 old-growth lowland forest sites, with no evidence of significant human disturbance, located in western, central-eastern and southern Amazonia (Fig 1 and SI Tables 2 and 3).

References:

-Brum, M. et al. Hydrological niche segregation defines forest structure and drought tolerance strategies in a seasonal Amazon forest. J. Ecol. 1–16 (2018). doi:10.1111/1365-2745.13022
-Barros, F. V. et al. Hydraulic traits explain differential responses of Amazonian forests to the 2015 El Nino-induced drought. New Phytologist, (2019).
-Bittencourt, P. R. L. et al. Amazonia trees have limited capacity to acclimate plant hydraulic properties in response to long-term drought. Glob. Chang. Biol. 26, 3569–3584 (2020).
-ForestPlots.net et al. Taking the pulse of Earth ' s tropical forests using networks of highly. 260, (2021).
-Lopez-Gonzalez, G., Lewis, S. L., Burkitt, M., Baker, T. R. & Phillips, O. L. ForestPlots.net Database. (2009). Available at: www.forestplots.net. (Accessed: 1st September 2018)
-Lopez-Gonzalez, G., Lewis, S. L., Burkitt, M. & Phillips, O. L. ForestPlots.net: A web application and research tool to manage and analyse tropical forest plot data. J. Veg. Sci. (2011). doi:10.1111/j.1654-1103.2011.01312.x

Sampling strategy

At each site, our sampling was focussed on the most dominant adult canopy and sub-canopy tree species, with sampling effort varying from 7 to 25 species which represents between 14% and 75% of the total basal area (SM Table 3). In total the sampled species account for ~24% of total Amazon tree biomass (Fauset et al., 2015) and spans a broad variety of life-history strategies (SM Fig 3). On average, we sampled 3 individuals per species per plot. Whenever possible, we prioritised sampling individuals of similar sizes and light exposure within a given species. Sites where less than 30% of the total basal area was sampled (ALP1, ALP2, SUC, CAX, MAN) are hyperdiverse forests and lack the clear dominance structure by a few species observed in less diverse plots (e.g. in the southern Amazon NVX site, the seven species sampled account for >50% of the basal area). Previous work by Barros et al. (2019), showed that the MAN site, despite having the lowest sampled basal area of all sites (~14%) is representative of the broader floristic community, as incorporating a broader array of species-level hydraulic trait data did not significantly change community-weighted mean (CWM) values. The same study found that mean species values are not likely to differ from community mean values if: (1) Species dominance is not driven by a few species, (2) traits have low dispersion around the mean (ie. low standard deviation compared the mean) and (3) traits are randomly distributed across species dominance. For the other 4 sites for which sampled coverage was less than 30%, these criteria are generally satisfied (e.g. cumulative dominance of the 5 most dominant species at ALP-1 is 27.9%, ALP-1 26.2%, SUC 15.0% and CAX 10.7% (SM Table 3), standard deviation of ψ50 is between 39 and 43% of the mean value at each site and there is no relationship between species dominance and hydraulic traits (SM Table 3). Thus, community-mean trait values for the 11 sites are likely to well represent the broader unsampled community of trees.

Reference:
-Fauset, S. et al. Hyperdominance in Amazonian forest carbon cycling. Nat. Commun. 6, 1–9 (2015).
-Barros, F. V. et al. Hydraulic traits explain differential responses of Amazonian forests to the 2015 El Nino-induced drought. New Phytologist, (2019).

Data collection

Plant material from the top canopy (or highest position reachable) was obtained by a tree climber using a telescopic scissor. During the wet season, immediately after collection, basal portions of branches were wrapped with a wet cloth and branches were placed in a humidified opaque plastic bag to avoid desiccation during transport. Bags were sealed and carried to the field station for determination of xylem vulnerability curves, branch wood density and leaf mass per area. For samples not collected during predawn (but always early morning), branches were placed in a bucket, re-cut under water, covered with an opaque plastic bag and left to rehydrate for at least 5 hours before determination of vulnerability curves. Detailed information about determinations of xylem vulnerability curves is provide in: Xylem embolism resistance (ψ50) in methods section. During the dry season, 3-6 leaves per individual were collected from top canopy and eaf water potential was measured with a pressure chamber (PMS 1505D and PMS 1000, PMS instruments).

Timing and spatial scale

For each site on which data collection was carried out, plant material to construct vulnerability curves and to measure branch wood density and leaf mass per area was undertaken during the wet season, when forests were maximally hydrated. Branches were harvested during predawn or very early in the morning, to capture a fully hydrated starting point (i.e in the vulnerability curve). SI Table 8 shows the sampling periods for all the plant traits evaluated in this study and SI Table 9 displays the number of species sampled per trait. Minimum in situ leaf water potential was measured from 11:00-2:30 in the peak of the dry season, except for aseasonal forests, which have no climatological dry season (monthlyprecip < 100mm) (Extended Data Fig. 2).

Data exclusions

Forest plots ALP-01 and ALP-02 (Plot codes from ForestPlot.net) have mixed soil types. To avoid bias due to soil type differences within tree communities, we used preferred plot views "ALP-01 poorly drained sandy clay" and "ALP-02 Shapajal clay soils" to calculate species dominance and carry out our data collection. These preferred plot views only include subplots, which total area is 0.48 ha (ALP-01) and 0.44 ha (ALP-02) that have the same soil type.

Before computing ψ50 per species per plot, we excluded all the branches that psi of maximum air discharge (AD) was less negative than - 2 Mpa, since no clear plateaus were detected. The maximum AD is the reference point for the calculation of the percentage of embolism formation and incorrect maximum AD will result in less negative ψ50 (Pereira et al., 2016; Trabi et al., 2021). Besides, we excluded from the analysis species which presented incomplete vulnerability curves (e.g. data points did not reach the plateau of maximum percentage air discharge). The exception of this criteria were applied for two species where was not possible to measure leaf water potential lower than -2 MPa using the pressure pump due to leaf drop. These two species (ALP2- Simarouba amara and TAM- Cedrelinga catenaeformis) were kept in the analyses because they all branches had the same pattern and no water potential measured was more negative than -2 MPa.

KEN1 and KEN1 plots were excluded from all forest dynamics analyses due to a fire event that occurred in the region in 2004 (Araujo-Murakami et al., 2014) and may still be affecting biomass accrual.

References:
-Lopez-Gonzalez, G., Lewis, S. L., Burkitt, M., Baker, T. R. & Phillips, O. L. ForestPlots.net Database. (2009). Available at: www.forestplots.net. (Accessed: 1st September 2018)
-Lopez-Gonzalez, G., Lewis, S. L., Burkitt, M. & Phillips, O. L. ForestPlots.net: A web application and research tool to manage and analyse tropical forest plot data. J. Veg. Sci. (2011). doi:10.1111/j.1654-1103.2011.01312.x

-Pereira, L., Bittencourt, P.R., Oliveira, R.S., Junior, M.B., Barros, F.V., Ribeiro, R.V. and Mazzafera, P. Plant pneumatics: stem air flow is related to embolism–new perspectives on methods in plant hydraulics. New Phytologist, 211(1), pp.357-370 (2016).
-Trabi C.L., et al. A User Manual to Measure Gas Diffusion Kinetics in Plants: Pneumatron Construction, Operation, and Data Analysis. Frontiers in Plant Science 12, (2021)
-Araujo-Murakami, A. et al. The productivity, allocation and cycling of carbon in forests at the dry margin of the Amazon forest in Bolivia. Plant Ecol. Divers. 7, 55–69 (2014)

Reproducibility

All details are described in the Methods session to allow vulnerability curves, measurements of minimum in situ leaf water potential, branch wood density and leaf mass per area to be reproduced. For each of these measures, sample collection was performed, on average, 3 times per species per site (on average 3 individuals per species).
To best ensure comparability between data it is important to keep standardised as much as possible the period of data collection and criteria for branch selection.

Randomization

Randomization was not relevant to our study since we evaluated relationships between forest dynamics metrics, vegetation traits and climatic factors across clusters of Amazonia forests individually through bivariate regression models. To calculate cluster mean values of forest dynamics metrics, we weighted each plot within a cluster by the product of plot monitoring length and the square root of plot area.

Blinding

Blinding was not relevant to our study.

Did the study involve field work? ☒ Yes ☐ No

Field work, collection and transport

Field conditions

Plant traits sampled collection was carried out in 8 sites in the western and southern Amazon, with mean annual precipitation regime and mean annual temperature varying from 1126 to 2880 mm and 23.4 to 26.3 °C across sites. Data from Central-eastern Amazon sites (TAP, MAN and CAX) were obtained from Brum et al. (2018), Barros et al. (2019) and Bittencourt et al. (2020), which follows the same methodology used in this present study. Site environmental characteristics are shown in supplementary material table 2.

References:
-Brum, M. et al. Hydrological niche segregation defines forest structure and drought tolerance strategies in a seasonal Amazon forest. J. Ecol. 1–16 (2018). doi:10.1111/1365-2745.13022
-Barros, F. V. et al. Hydraulic traits explain differential responses of Amazonian forests to the 2015 El Nino-induced drought. New Phytologist, (2019).
-Bittencourt, P. R. L. et al. Amazonia trees have limited capacity to acclimate plant hydraulic properties in response to long-term drought. Glob. Chang. Biol. 26, 3569–3584 (2020).

Location

Samples were collected in terra-firme lowland forest plots (<400 m.a.s.l. altitude), being all plots part of RAINFOR permanent inventory plot network. Figure 1 shows a map with all site locations.
SUC (Sucusari) - Iquitos, Maynas, Peru 3°15'S, 72°54'W
ALP1 and ALP-2 (Allpahuayo) - Iquitos, Maynas, Peru 3°56'S, 73°25'W
TAM (Tambopata) - Puerto Maldonado, Madre de Dios, Peru 12°49'S, 69°16'W
FEC (Fazenda Experimental Catuaba) - Senador Guiomard, Acre, Brazil 10°4'S, 67°37'W
KEN1 and KEN2 (Kenia) - Ascensión de Guarayos, Santa Cruz, Bolivia 16°1'S, 62°43'W
NXV (VCR) - Fazenda Vera Cruz, Nova Xavantina, Mato Grosso, Brazil 14°49'S, 54°84'W
Central-eastern Amazon forests data obtained from Brum et al. (2018), Barros et al. (2019) and Bittencourt et al. (2020).

References:
-ForestPlots.net et al. Taking the pulse of Earth ' s tropical forests using networks of highly. 260, (2021).
-Brum, M. et al. Hydrological niche segregation defines forest structure and drought tolerance strategies in a seasonal Amazon forest. J. Ecol. 1–16 (2018). doi:10.1111/1365-2745.13022
-Barros, F. V. et al. Hydraulic traits explain differential responses of Amazonian forests to the 2015 El Nino-induced drought. New Phytologist, (2019).
-Bittencourt, P. R. L. et al. Amazonia trees have limited capacity to acclimate plant hydraulic properties in response to long-term drought. Glob. Chang. Biol. 26, 3569–3584 (2020).

Access & import/export

The permits for sample collection in each site were granted in the name of Prof. Dr. David R. Galbraith/Tremor Project and conceded by the following national responsible authorities:

- SUC - Gestión Sostenible del Patrimonio Forestal y de Fauna Silvestre (SERFOR). nº121-2016-GGR-ARA-DEFFS-DER, Date: 29/11/2016;
- ALP - Dirección de Gestión de las Áreas Naturales Protegidas (SERNANP). nº 073-2017-SERNANP-RNAM-J, Date: 18/11/2016;
- TAM - Dirección de Gestión de las Áreas Naturales Protegidas (SERNANP). nº 039-2016-SERNANP-RNTAMB-PRD, Date: 08/09/2016;
- FEC - Instituto Chico Mendes de Conservação da Biodiversidade (ICMBio), Sistema de Autorização e Informação em Biodiversidade (SISBIO) - Número: 57821-1, Date: 07/03/2017;
- KEN - Ministerio de Medio Ambiente y Agua - Viceministerio de Medio Ambiente, Biodiversidad y Cambios Climáticos. CAR-MMAYA/VMABCCGDF/DGBAP/MEY nº 0198/2017, Date: 27/03/2017;

NXV site is located in a private area and, apart from the farm's owner, no other permit is needed. The owner of Vera Cruz farm granted the collection permit in the name of Prof. Dr. Beatriz Schwantes Marimon.

Disturbance

Our sampling did not cause significant disturbances on the plot. Top tree canopy were assessed by climbers using climbing gears and terminal branches were cut with telescopic scissors.

Reporting for specific materials, systems and methods

We require information from authors about some types of materials, experimental systems and methods used in many studies. Here, indicate whether each material, system or method listed is relevant to your study. If you are not sure if a list item applies to your research, read the appropriate section before selecting a response.

Materials & experimental systems

n/a	Involved in the study
☒	☐ Antibodies
☒	☐ Eukaryotic cell lines
☒	☐ Palaeontology and archaeology
☒	☐ Animals and other organisms
☒	☐ Human research participants
☒	☐ Clinical data
☒	☐ Dual use research of concern

Methods

n/a	Involved in the study
☒	☐ ChIP-seq
☒	☐ Flow cytometry
☒	☐ MRI-based neuroimaging
